# Dual‐Terminal Molecular Strategy for Robust and Reversible Supramolecular Adhesion

**DOI:** 10.1002/advs.202511818

**Published:** 2025-08-29

**Authors:** Shiru Wang, Liang Meng, Feng Li, Yuru Wang, Yongri Liang, Guangming Lu, Keju Sun, Yingdan Liu, Jingyue Yang

**Affiliations:** ^1^ State Key Laboratory of Metastable Materials Science and Technology Nano‐bio‐technology Key Lab of Hebei Province Applying Chemistry Key Lab of Hebei Province Yanshan University Qinhuangdao 066004 P. R. China; ^2^ Key Laboratory of Green Chemistry & Technology of Ministry of Education College of Chemistry Sichuan University 29 Wangjing Road Chengdu Sichuan 610064 P. R. China; ^3^ Center of Advanced Structural Materials State Key Lab of Metastable Materials Science and Technology School of Materials Science and Engineering Yanshan University No. 438 West Hebei Avenue Qinhuangdao Hebei 066012 P. R. China; ^4^ State Key Laboratory of Advanced Marine Materials Ningbo Institute of Materials Technology and Engineering Chinese Academy of Sciences Ningbo 315201 P. R. China

**Keywords:** hydrophobic substrate adaptability, instantaneous adhesion, minimalist modular supramolecular adhesive, robust adhesion strength, sustainability

## Abstract

Achieving strong yet reversible adhesion via minimalist molecular design remains a critical challenge for next‐generation supramolecular materials. Here, a dual‐end modular adhesion strategy is presented based on a small organic molecule incorporating carboxylic acid and triphenylphosphonium terminals linked by a flexible alkyl spacer. This design enables synergistic noncovalent interactions—including hydrogen bonding, dipole–dipole interactions, and electrostatic forces—to construct a thermally reconfigurable supramolecular network. Upon mild heating, the system transitions from ordered to amorphous states, facilitating dynamic cohesion and interfacial adaptability across both hydrophilic and hydrophobic substrates. The resulting adhesive achieves high lap‐shear strength (up to 4.6 MPa on polyethylene terephthalate (PET)), rapid curing, and exceptional resistance to solvents, humidity, and low temperatures. Moreover, it enables fully reversible adhesion and closed‐loop recyclability. Combined experimental characterizations and molecular simulations reveal how the interplay of molecular architecture and noncovalent synergy governs adhesion performance. This work provides a generalizable framework for the design of sustainable, programmable supramolecular adhesives.

## Graphical abstract

A minimalist supramolecular adhesive derived from a dual‐terminal molecule bearing carboxylic acid and triphenylphosphonium groups achieves strong, reversible adhesion through synergistic noncovalent interactions. The system exhibits rapid curing, broad substrate compatibility, and exceptional stability under solvents, humidity, and extreme temperatures, with a high adhesion strength of 4.6 MPa on polyethylene terephthalate (PET).

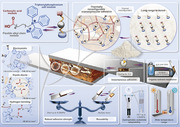

## 1 Introduction

The majority of contemporary high‐performance adhesives are built upon crosslinked polymer matrices,^[^ ^1^ ^]^ which rely on covalent networks to deliver excellent adhesion and mechanical robustness.^[^ ^2^, ^3^ ^]^ While these densely packed structures enable impressive bond strength, their irreversible nature introduces significant limitations—including difficult recycling, poor reprocessability,^[^ ^4^ ^]^ and low adaptability under variable environmental conditions. Moreover, prolonged curing times and reduced performance at low temperatures further hinder their applicability, especially in fast‐paced or sustainability‐driven industries.^[^ ^5^ ^]^ These limitations highlight a growing demand for next‐generation adhesives that combine strong, rapid, and reversible adhesion with structural simplicity and environmental resilience.

In contrast to traditional systems, supramolecular adhesives, which are formed through dynamic noncovalent interactions, such as hydrogen bonding, π–π stacking, electrostatic interactions, π‐cation interactions, and dipole–dipole interactions, enable reversibility, mild processing, and stimulus‐responsiveness.^[^ ^6^ ^]^ However, achieving simultaneous strength and sustainability remains a central challenge.^[^ ^7^, ^8^ ^]^ Most supramolecular systems based on low‐molecular‐weight motifs exhibit insufficient cohesive and interfacial adhesion due to the limited number and strength of intermolecular interactions.^[^ ^9^ ^]^ Strategies involving dynamic covalent bonds, such as disulfide and Diels‐Alder bonds, improve reversibility but often compromise mechanical integrity upon repeated use.^[^ ^10^ ^]^ A more promising direction lies in the modular design of small‐molecule adhesives that synergistically integrate multiple noncovalent interactions to enhance adhesion.^[^ ^11^ ^]^

To date, representative efforts in this direction include crown ether–based architectures^[^ ^12^ ^]^ and the integration of ionic liquids into adhesive matrices.^[^ ^13^ ^]^ Nonetheless, the complexities associated with the rational design of molecular models, their synthesis, and the intricate understanding of the adhesive mechanism have served as significant barriers to the advancement of this strategy.^[^ ^14^, ^15^ ^]^ The development of such minimalist yet effective architectures remains underexplored.

**Figure 1:**
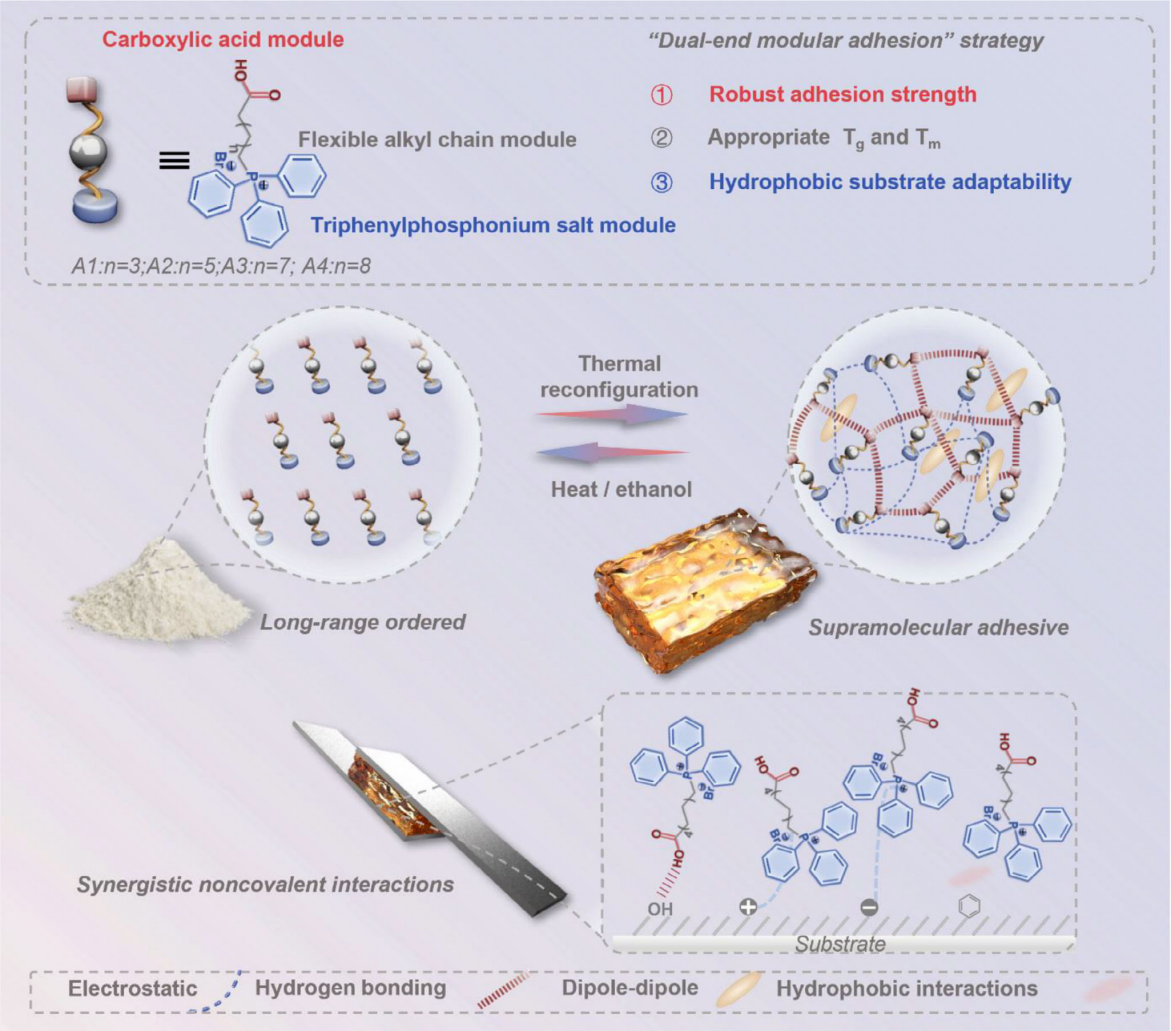
Schematic illustration of dual‐terminal synergy driving dynamic network formation in the minimalist modular supramolecular adhesive.

Herein, we introduce a dual‐end modular adhesion strategy to construct a minimalist, single‐component supramolecular adhesive (**Figure** **1**). This architecture features two complementary terminals: a carboxylic acid group that enables directional hydrogen bonding, and a triphenylphosphonium salt moiety that provides strong affinity via dipole–dipole interactions and electrostatic interactions. These functional terminals are connected by a flexible alkyl linker, facilitating thermal responsiveness and mobility. Inspired by the hydrogen‐bond‐rich motifs of poly(acrylic acid),^[^ ^16^ ^]^ but fundamentally restructured into a reconfigurable small‐molecule framework, this adhesive exhibits thermally induced supramolecular reorganization from ordered to amorphous states—providing both mechanical integrity and recyclability. This design achieves exceptional adhesion strength (up to 4.6 MPa on polyethylene terephthalate (PET)), broad substrate compatibility, and high environmental tolerance, while enabling rapid curing and multi‐cycle reusability. Through combined experimental analysis and molecular simulations, we elucidate how dual‐terminal synergy governs the dynamic assembly of noncovalent networks, providing a robust and generalizable platform for future sustainable adhesive systems.

## 2 Results and Discussion

### 2.1 Modular Design and Assembly

**Figure 2:**
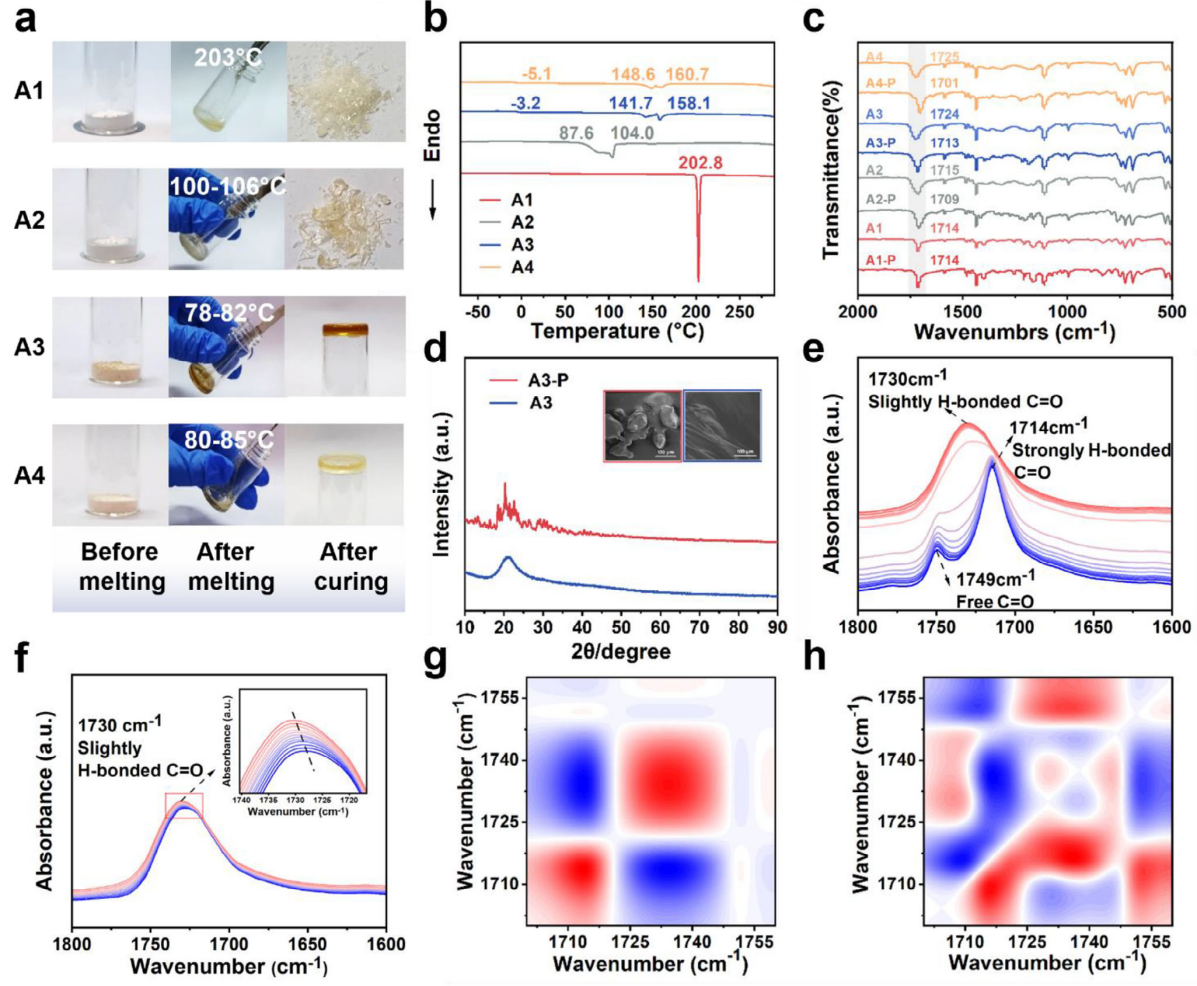
Thermal reconfiguration and structural evolution of dual‐end modular adhesives. a) Photographs of **A1**, **A2**, **A3**, and **A4** during thermal reconfiguration, illustrating their ability to liquefy and subsequently resolidify. b) DSC curves of four cured adhesives. c) FT‐IR spectra of powdered and cured forms (“P” indicates powdered state) of four compounds. d) XRD diffractogram of **A3** before and after thermal reconfiguration (“P” indicates powdered form), inset shows SEM images of **A3** in powdered and reconfigured states. e, f). Temperature‐dependent FT‐IR spectra of **A3** during heating (30–150 °C) and subsequent cooling (150–30 °C) at 10 °C intervals. g,h) 2D COS synchronous and asynchronous spectra generated from g, wherein red and blue colors are defined as positive and negative intensities, respectively.

Given the critical role of alkyl linker segments in modulating the thermomechanical properties of molecular adhesives,^[^ ^17^ ^]^ a series of dual‐end modular adhesives (BrPPh_3_‐R‐COOH, **A1–A4**) featuring tailored alkyl chain lengths were synthesized via a straightforward, single‐step reaction between readily accessible triphenylphosphine and the corresponding brominated carboxylic acids. Their structures were confirmed by nuclear magnetic resonance (NMR) spectroscopy and high‐resolution electrospray ionization mass spectrometry (HR‐ESI‐MS) (Scheme S1 and Figures S1–S6, Supporting Information). As depicted in **Figure** **2**a, all four compounds exhibited non‐viscous, loosely packed powder forms at ambient conditions. Interestingly, their melting points followed a clear trend as alkyl chain length increased, initially decreasing and then slightly rising: **A3** displayed the lowest melting point (≈80 °C), while **A4** required slightly higher temperature, and **A1** and **A2** demanded significantly higher temperatures (>200 °C and >100 °C, respectively). This suggests that **A3** possesses a heightened temperature responsiveness compared to the other three compounds.

Upon thermal reconfiguration, distinct variations in physical behavior emerged across the series. When heated, all compounds demonstrated viscoelasticity and could be drawn into filaments. However, after curing, **A1** solidified into a brittle, non‐viscous material, and **A2** also exhibited fragility. Notably, **A3** and **A4** retained remarkable tenacity in their amorphous solid states, indicating the formation of a thermally reconfigurable supramolecular network. These observations were corroborated by comprehensive structural and thermal analyses, including differential scanning calorimetry (DSC), Fourier‐transform infrared (FT‐IR) spectroscopy, X‐ray diffraction (XRD), and scanning electron microscopy (SEM). Specifically, thermal reconfiguration substantially modified the behavior of **A3**, lowering its glass transition temperature (T_g_) to −3.2 °C and increasing its melting temperature to 158.1 °C, compared to 78–82 °C before treatment. **A4** displayed analogous transitions. By contrast, **A2** retained a melting temperature comparable to its powdered form, and **A1** maintained a sharp crystallization melting peak at 202.8 °C, indicating persistent crystallinity (Figure 2b). FT‐IR analysis before and after thermal treatment further illuminated these transformations. The infrared spectrum of **A1** remained unchanged; the C═O stretching bond of **A2** exhibited a minor shift; whereas **A3** and **A4** underwent significant shifts, signaling the formation of reconfigurable supramolecular networks, a conclusion consistent with XRD evidence (Figure 2c; Figure S13, Supporting Information). For **A3**, XRD and SEM revealed that the powdered material initially displayed sharp diffraction peaks and a polycrystalline morphology, reflecting long‐rang order). After thermal reconfiguration, a broad diffuse XRD peak emerged, and SEM images showed a dense, flattened surface with amorphous character, indicative of a transition to a disordered but cohesive supramolecular network (Figure 2d).

To probe the molecular‐level evolution during heating, variable‐temperature FT‐IR spectroscopy was performed (Figure 2e). **A3** initially exhibited two distinct C═O absorption bands at 1714 and 1749 cm^−1^, corresponding to strongly hydrogen‐bonded and free carboxyl groups, respectively. As temperature increased, the 1714 cm^−1^ peak and 1749 cm^−1^ peak gradually diminished successively, while the 1730 cm^−1^ band emerged, signifying the reorganization of slightly hydrogen‐bonded C═O species. Upon cooling, the 1730 cm^−1^ band underwent a red shift, suggesting the reinforcement of the newly formed supramolecular network (Figure 2f). Complementary synchronous and asynchronous 2D correlation spectroscopy (2DCOS) confirmed the sequential transition of 1714 → 1730 → 1749 cm^−1^, underscoring that hydrogen‐bonded carboxylic acid dissociation and free group reorganization underlie the reduction of molecular ordering and the emergence of a stable yet dynamic adhesive network (Figure 2g,h).^[^ ^18^ ^]^ Thermal stability assessments using thermogravimetric analysis (TGA) demonstrated excellent material robustness, with all compounds showing <4.2% mass loss when heated up to 280 °C (Figure S14, Supporting Information), confirming no degradation during the hot‐melt process (≈150 °C). Rheological measurements on thermally reconfigured **A3** further revealed its liquid‐like characteristic, setting the foundation for subsequent exploration of its adhesive performance (Figure S15, Supporting Information).

### 2.2 Adhesion performance across diverse substrates

**Figure 3:**
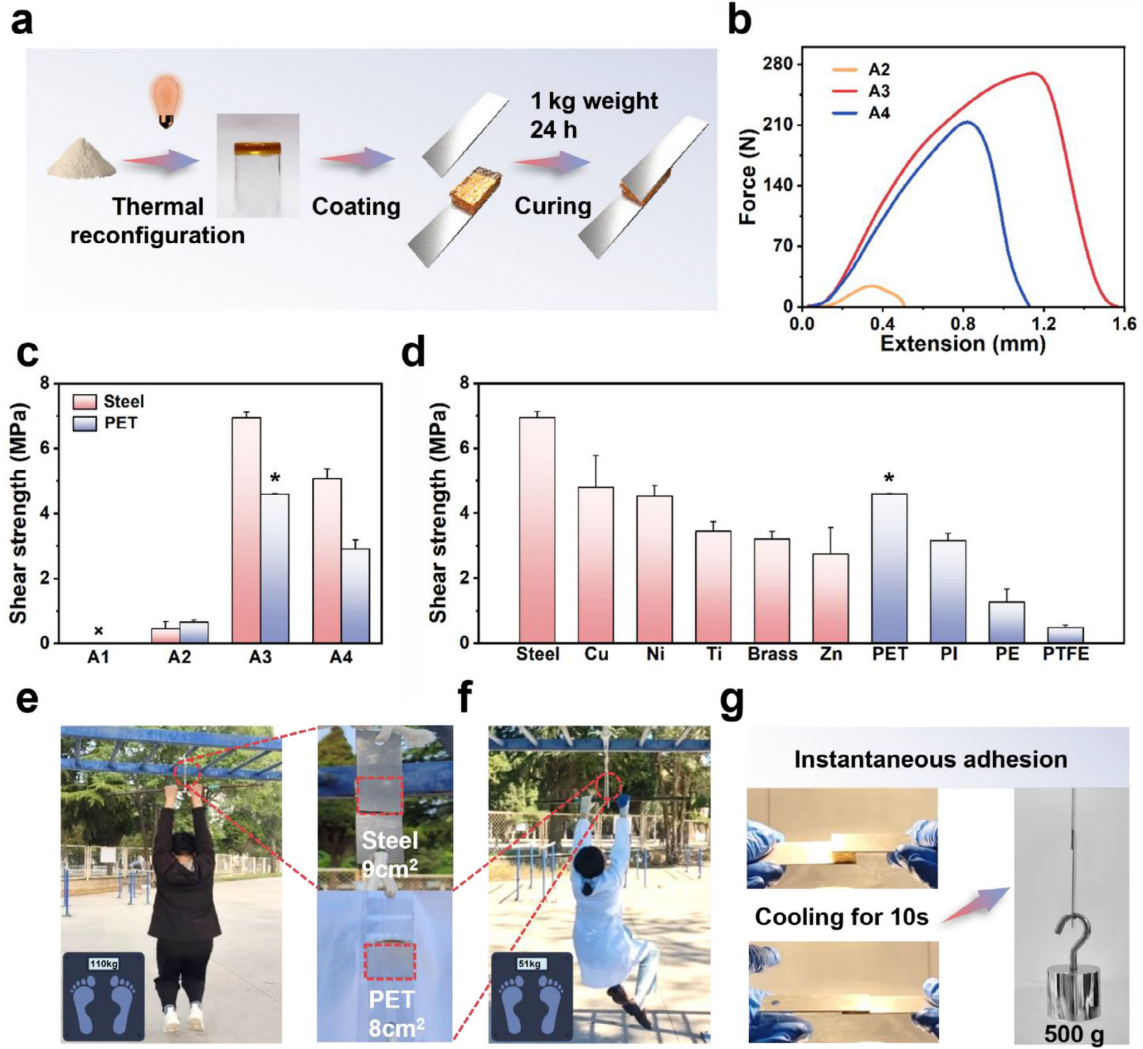
Adhesion performance and load‐bearing capacity of dual‐end modular adhesives. a) Schematic of the adhesion procedure. b, c) Lap‐shear test curves and corresponding adhesion strengths of dual‐end modular adhesives. d) Adhesion strengths of **A3** on various substrates under ambient conditions (“*” indicates substrate deformation). e) Macroscopic load‐bearing demonstration: **A3** bonded on Steel (9 cm^2^ adhesion area) sustaining 110 kg. f) Macroscopic load‐bearing demonstration: **A3** bonded on PET (8 cm^2^ adhesion area) sustaining 51 kg. g) Macroscopic demonstration of the instantaneous adhesion of **A3**. All data were presented as mean ± SD (n =3–5 independent samples).

Following the successful synthesis of the dual‐end modular adhesives, their adhesive performance was investigated on both hydrophilic (Steel) and hydrophobic (PET) substrates through lap‐shear testing. Thermal reconfiguration was achieved using a near‐infrared lamp (≈150 °C, 3–5 min), followed by curing at ambient temperature under a pressure of 1 kg (**Figure** **3**a).

As depicted in Figure 3b,c, **A3** demonstrated the highest adhesion strength in the series, achieving 6.95 MPa on Steel and 4.6 MPa on PET, thereby establishing itself as a high‐performance, single‐component supramolecular adhesive. Concurrently, **A4** exhibited adhesive strengths of 5.07 MPa on Steel and 2.91 MPa on PET, while **A2** remained below 1 MPa on both substrates. Due to its elevated melting point, **A1** posed significant challenges during application, preventing the reliable measurement of its adhesion strength. Furthermore, as shown in Figure S16 (Supporting Information), the analysis of the work of debonding revealed that **A3** exhibited a significantly higher value of 6075.52 N·m^−1^, in comparison to **A4** (2837.26 N·m^−1^) and **A2** (152.8 N·m^−1^), consistent with the superior tenacity observed in thermally reconfigured samples. The curing kinetics of **A3** were comprehensively evaluated under varying durations and environmental conditions. Optimal adhesion was achieved after a curing period of 24 h (Figure S17, Supporting Information), yet the system demonstrated excellent resilience under environmental stress, with adhesion strength decreasing by <20% even when curing humidity exceeded 60% (Figure S18, Supporting Information).

Encouragingly, **A3**, selected as the representative minimalist modular supramolecular adhesive, demonstrated superior adhesion not only on hydrophilic materials including steel (Steel), nickel (Ni), copper (Cu), titanium (Ti), brass, and zinc (Zn), where strengths exceeded 3 MPa, but also on traditionally challenging hydrophobic substrates such as PET, polyimide (PI), polyethylene (PE), and polytetrafluoroethylene (PTFE), which typically exhibit adhesion strengths below 0.5 MPa (Figure 3d; Tables S1 and S3, Supporting Information). Particularly notable was the high adhesion strength (4.6 MPa) achieved on PET, which led to the complete deformation of a 1 mm thick PET substrate during lap‐shear testing (Figure S19, Supporting Information). Adhesion strengths on PI and PE reached 3.16 and 1.27 MPa, respectively, which are remarkable values for these materials. To further demonstrate the robust adhesive capabilities of **A3**, macroscopic load‐bearing tests were conducted (Figure 3e,f; Videos S1 and S2, Supporting Information). A pair of Steel plates (9 cm^2^ bonded area) readily supported a 110 kg load, while bonded PET substrates (8 cm^2^) was able to sustain a load of 51 kg, exceeding 100 000 times the weight of the adhesive applied (0.5 g). Besides, **A3** demonstrated robust instantaneous adhesion: Steel samples cured for as little as 10 s were able to support a 500 g load with ease (Figure 3g; Video S3, Supporting Information).

### 2.3 Environmental Robustness and Operational Stability of the Modular Adhesive A3

**Figure 4:**
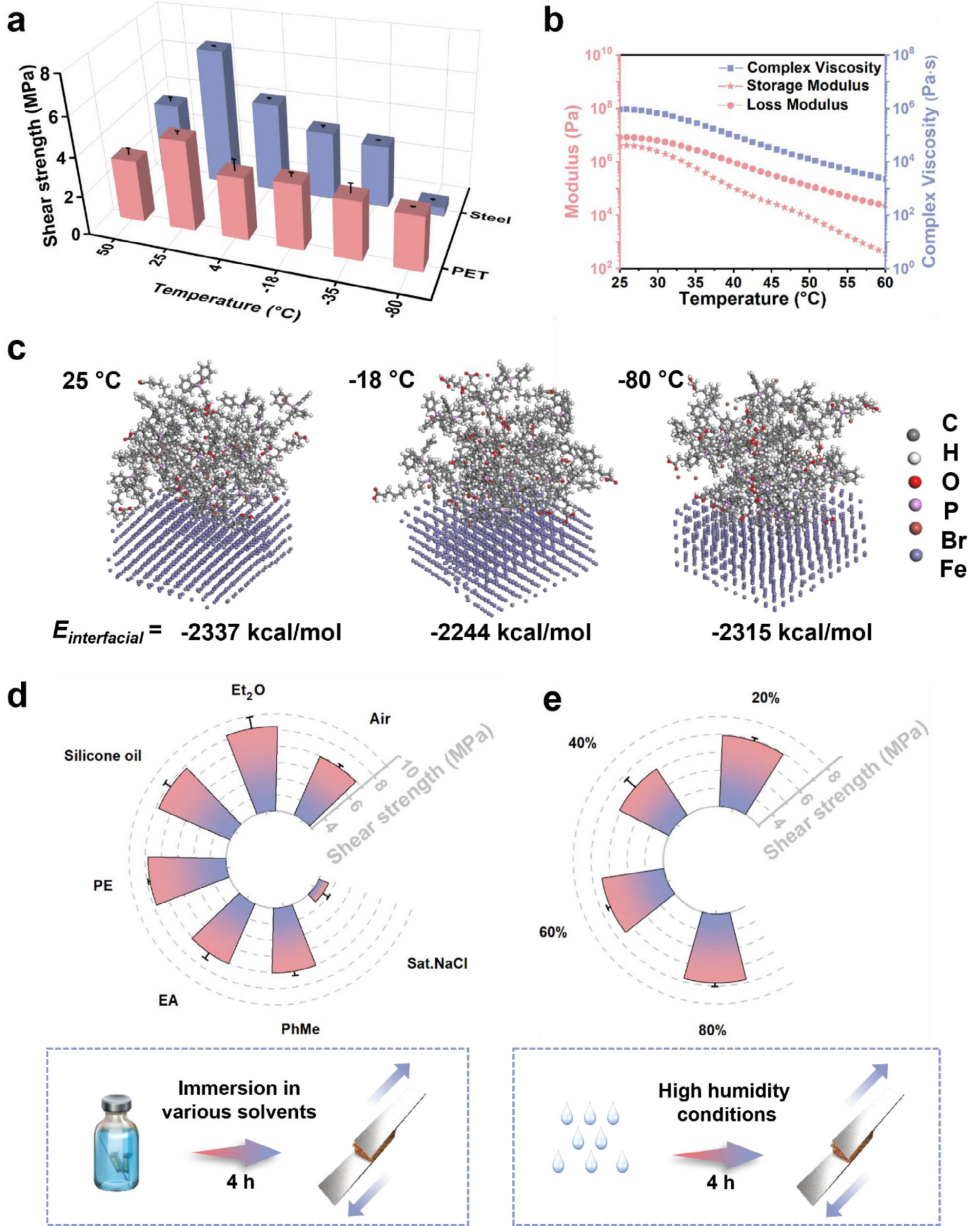
Environmental stability and multi‐scenario durability of modular adhesive **A3**. a) Temperature‐dependent adhesion strengths of modular adhesive **A3** on Steel and PET. b) Temperature‐dependent rheological characterization of modular adhesive **A3**. c) Interfacial adhesive energy (IAE) of **A3** on Fe at 25, −18, and −80 °C. d) Adhesion strength of **A3** after 4‐h immersion in various solvents on Steel. e) Adhesion stability of **A3** following 4‐h exposure to different humidity levels at room temperature. All data were presented as mean ± SD (n =3–5 independent samples).

Supramolecular adhesives are often highly sensitive to environmental stimuli, particularly temperature fluctuations, which can substantially alter their adhesive properties.^[^ ^7^, ^16^, ^18^ ^]^ To evaluate the thermal resilience of the dual‐end modular adhesive **A3**, lap‐shear tests were conducted across a wide temperature range. Cured samples were exposed for 4 h to 50, 25, 4, −18, −35, and −80 °C, respectively. As shown in **Figure** **4**a, **A3** maintained outstanding adhesion strength on PET, exceeding 3 MPa from 50 °C to −35 °C. Even at −80 °C, it retained a notable strength of 2.8 MPa, surpassing many commercial adhesives operating at room temperature. In contrast, a substantial decrease in adhesion strength was observed on Steel, plummeting to 0.42 MPa at −80 °C.

To further investigate the temperature effects, rheological measurements were performed under heating, and molecular dynamics (MD) simulations were conducted to calculate the interfacial adhesion energy (IAE) of **A3** on Fe at low temperatures. Rheological results indicated a marked decrease in both viscosity and modulus with increasing temperature, enhancing flowability and substrate wetting but reducing cohesive integrity (Figure 4b).^[^ ^19^ ^]^ MD simulations revealed relatively stable IAE across different temperatures, with values of −2337 kcal mol^−1^ at 25 °C, −2244 kcal mol^−1^ at −18 °C, and −2315 kcal mol^−1^ at −80 °C (Figure 4c; Table S2, Supporting Information). These findings imply that the sharp decrease in adhesion on Steel at −80 °C is not simply due to weakened intermolecular forces but is more likely attributed to thermal mismatch‐induced stress concentration at the adhesive–substrate interface, arising from differential thermal expansion and mechanical property changes.^[^ ^17^ ^]^

Beyond thermal performance, the adhesive also demonstrated excellent chemical resistance. Conventional polymer‐based adhesives are prone to solvent infiltration, leading to adhesion failure.^[^ ^20^, ^21^ ^]^ In contrast, **A3** exhibited robust resistance against a wide range of organic solvents, including toluene (PhMe), ethyl acetate (EA), ethyl ether (Et_2_O), petroleum ether (PE), and silicone oil, after 4 h of immersion, even showing enhanced adhesion strength in most cases (Figure 4d). Although a slight decrease in performance was observed in aqueous NaCl solution, the adhesive retained a high strength of 3.65 MPa. For long‐term testing, samples were immersed for up to 12 months, with only Et_2_O causing bond failure after ≈2 months (Figure S20, Supporting Information), while all other solvent‐bonded samples remained intact throughout the testing period.

To simulate real‐world operational conditions, humidity tolerance was further assessed. Steel plates bonded with **A3** adhesive were exposed to a range of humidity conditions at room temperature for 4 h prior to testing. As illustrated in Figure 4e, the adhesive exhibited minimal sensitivity to humidity, maintaining stable performance across low to high humidity environments. This can be attributed to the formation of a dense, non‐porous interfacial adhesive layer post‐curing, which effectively prevents moisture penetration. The environmental stability was further validated by a long‐term load‐bearing test, in which a Steel sample (8 cm^2^ bonding area) held a 500 g weight for over 3 weeks in an environment with fluctuating humidity(60–90%)without bond failure (Figure S21, Supporting Information).

### 2.4 Reconfigurability and Closed‐Loop Recyclability

**Figure 5:**
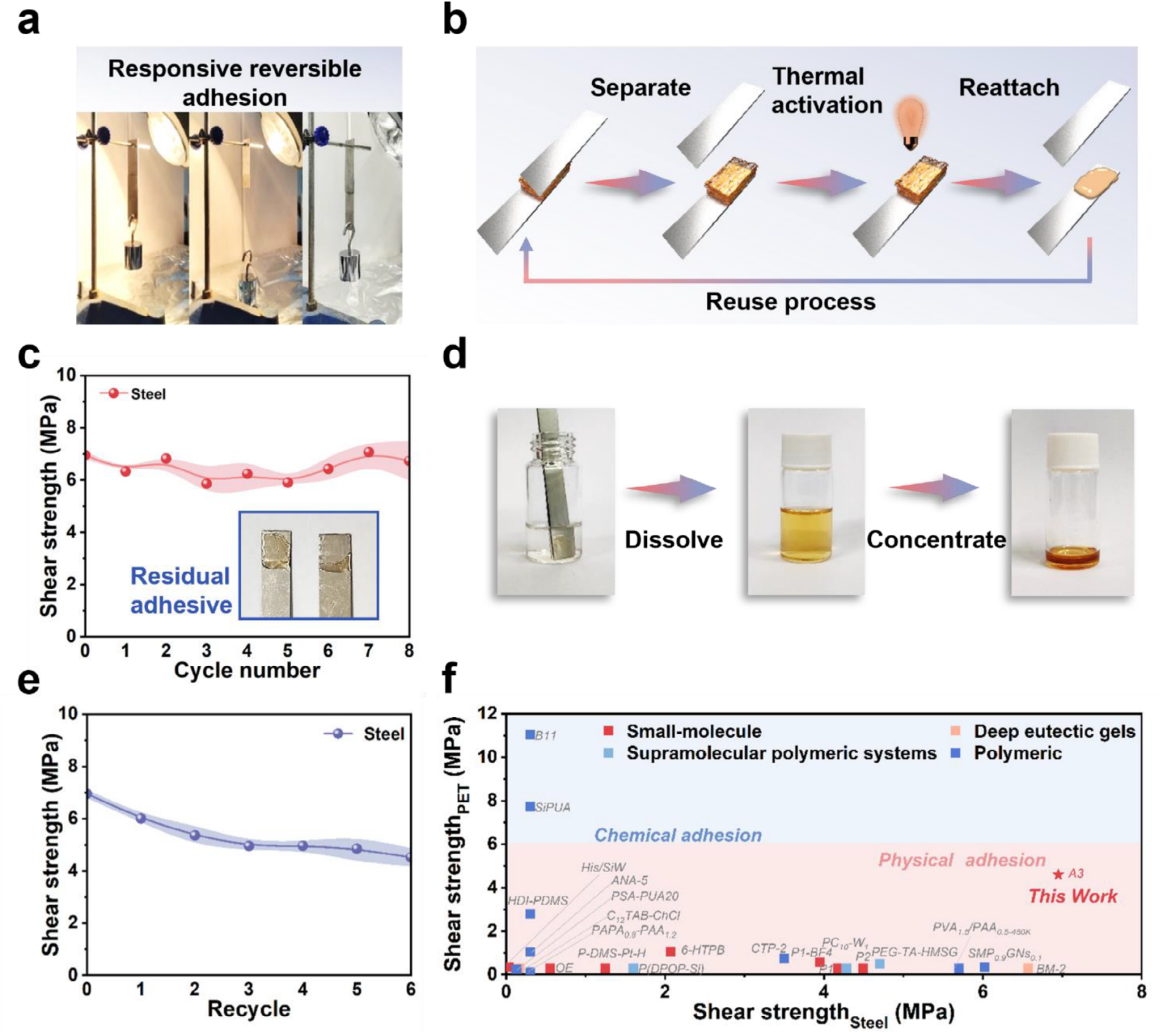
Reversible adhesion and closed‐loop recyclability of modular adhesive **A3**. a) Responsive reversible adhesion of **A3** under NIR irradiation. b) Schematic illustration of the reuse process of **A3**. c) Adhesion strength of **A3** on Steel after eight “attach‐separate‐reattach” cycles; inset shows residual adhesive on the Steel surface following lap‐shear testing. d) Closed‐loop recycling of **A3** via dissolution in alcohol and subsequent solvent evaporation. e) Adhesion strength of recycled **A3** on Steel after multiple recovery cycles. f) Comparison analysis of the adhesive strength of **A3** on PET and Steel versus reported adhesive systems.^[^ ^6^, ^11^, ^13^, ^26^, ^27^, ^28^ ^]^ All data were presented as mean ± SD (n =3–5 independent samples).

Reusability and recyclability are critical metrics for next‐generation adhesives, aligning not only with the demands of industrial sustainability but also with broader environmental imperatives. One inherent advantage of low‐molecular‐weight systems over conventional polymeric adhesives lies in their stimuli‐responsive and modular nature, which enables structural reconfiguration upon external triggers.^[^ ^8^, ^22^, ^23^ ^]^ As depicted in **Figure** **5**a and Video S4 (Supporting Information), a pair of Steel plates bonded with modular adhesive **A3** and loaded with a 500 g weight were exposed to a near‐infrared lamp. Within seconds, thermal activation induced adhesive softening and detachment. Remarkably, the same plates could be re‐adhered and recooled to room temperature, re‐establishing their load‐bearing capacity. This cycle was repeated eight times, with no detectable loss in adhesion strength or visible damage to the substrate surfaces, demonstrating excellent reusability and thermally triggered reversibility (Figure 5b,c).

An alternative method for reusing the modular adhesive **A3** involves recycling. As illustrated in Figure 5d, residual adhesive on the substrate was readily dissolved in environmentally benign ethanol and recovered by simple evaporation. The reclaimed material was reused in lap‐shear tests over six cycles. As shown in Figure 5e, the recycled adhesive retained approximately 65% of its initial adhesive strength, with the decline likely attributable to trace ethanol residues disrupting optimal supramolecular assembly. Supporting this, TGA revealed a 5.21% increase in mass loss during the initial heating ramp, corresponding closely to the residual ethanol content detected via ^1^H NMR spectrum (Figures S22 and S23, Supporting Information). Given the high density of hydrogen bonding motifs within **A3**, residual solvent molecules may be strongly retained within the supramolecular matrix, influencing performance recovery.

In a comparative context, the dual‐terminal modular adhesive **A3** markedly outperforms most state‐of‐the‐art supramolecular and polymer‐based adhesives across both hydrophilic and hydrophobic substrates. As shown in Figure 5f and Figure S24 (Supporting Information), it delivers exceptional adhesion strength on Steel, PET, and even inert PTFE, surpassing many polymer‐based systems (Table S3, Supporting Information) despite its remarkably low molecular weight (513.45 g mol^−1^). Notably, **A3** achieves adhesion strength on Steel that are on par with high‐performance polymer adhesives, thereby overcoming a persistent challenge in the field—namely, the trade‐off between low molecular weight and mechanical robustness in supramolecular systems. Achieving strong adhesion on low‐energy substrates such as PET typically necessitates specific chemical anchoring strategies.^[^ ^24^, ^25^ ^]^ In contrast, **A3**, establishes robust interfacial adhesion through purely physical and noncovalent mechanisms, delivering superior performance on both PET and PTFE, and outcompeting a broad range of platforms, including small‐molecule adhesives,^[^ ^6^, ^11^, ^13^, ^26^ ^]^ deep eutectic gels,^[^ ^6a,f^ ^]^ polymeric^[^ ^27^ ^]^ and supramolecular polymeric systems.^[^ ^28^ ^]^ A comprehensive comparison in Table S4 (Supporting Information) highlights the unique advantages of **A3** in terms of PET adhesion strength, reusability, curing time, substrate versatility, and solvent resistance, underscoring its superior performance among state‐of‐the‐art small‐molecule adhesives. These findings underscore the multifunctional superiority and broad substrate applicability of the dual‐terminal modular adhesion strategy, highlighting its promise as a versatile and recyclable platform for next‐generation intelligent adhesion technologies.

### 2.5 Noncovalent Interaction Mechanism Governing Modular Adhesion

**Figure 6:**
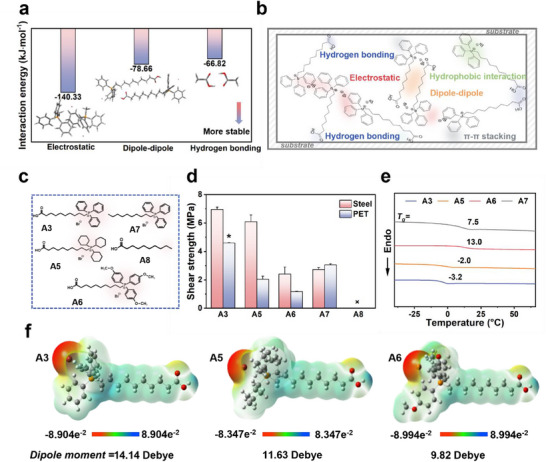
Molecular‐level insights into cohesion and adhesion mechanisms. a) DFT calculation results for electrostatic, dipole–dipole, and hydrogen bonding interactions. b) Schematic illustration of the proposed mechanism underlying cohesion and adhesion for **A3**. c) Chemical structures of control compounds **A3**, **A5**–**A8**. d) Lap‐shear adhesion strengths of **A3**, **A5**–**A8** on Steel and PET substrates. e) DSC curves of **A3**, **A5** and **A6** adhesives. f) Surface electrostatic potential maps and calculated dipole moments for the lowest energy conformations of **A3**, **A5**, and **A6**. All data were presented as mean ± SD (n =3–5 independent samples).

To elucidate the molecular basis for the exceptional adhesive strength of modular adhesive **A3**, we investigate the supramolecular interactions that govern its structural cohesion and interfacial binding. The high performance of **A3** is attributed to a finely balanced interplay between cohesion and adhesive forces, enabled by the supramolecular assembly of monomers into a thermally reconfigurable network through diverse noncovalent interactions. To quantify the contributions of these interactions, density functional theory (DFT) calculations were performed to assess the binding energies between representative molecular fragments of **A3** (**Figure** **6**a). The optimized geometries and calculated interaction energies revealed significant binding strengths for electrostatic (−140.33 kJ mol^−1^), dipole–dipole (−78.66 kJ mol^−1^), and hydrogen bonding (−66.82 kJ mol^−1^) interactions. These potent forces play a critical role in driving molecular assembly and maintaining the structural integrity of the adhesive under diverse conditions. A schematic overview of the cohesive and interfacial adhesion pathways is shown in Figure 6b, highlighting the cooperative contribution of abundant, high‐strength noncovalent interactions to the adaptability and surface compatibility of the modular adhesive system.

The molecular geometry of the dual‐end modular adhesives—including the triphenylphosphonium salt moiety, its substituents, and the carboxylic acid terminal—was further examined to delineate their specific roles in supramolecular assembly. Control experiments affirmed the importance of both components (Figure 6c). Structural modifications on the phosphonium salt moiety, such as cyclohexyl (**A5**) and methoxybenzyl (**A6**) substitution, retained moderate adhesive strength, indicating the resilience of the modular framework (Schemes S2 and S3 and Figures S7–S10, Supporting Information). Elimination of the carboxyl group (**A7**) significantly weakened adhesion, and removal of the phosphonium salt (**A8**) led to a complete loss of adhesive capability (Scheme S4 and Figures S11 and S12, Supporting Information). These observations underscore the synergistic role of the dual‐terminal design in facilitating efficient supramolecular network formation and robust adhesion. Lap‐shear tests revealed that **A5** and **A6** retained adhesion strengths above 2 MPa (Steel) and 1 MPa (PET), validating the preservation of dominant intermolecular interactions despite structural perturbations (Figure 6d). Nevertheless, the T_g_ of **A5**, **A6**, and **A7** increased to −2.0, 13.0, and 7.5 °C, respectively, compared to −3.2 °C of **A3**, indicating reduced low‐temperature flexibility and adhesive adaptability (Figure 6e). DFT‐based dipole moment calculations further revealed that **A3** exhibited the highest dipole moment (14.14 D), enhancing intermolecular interactions, whereas **A5** and **A6** show reduced dipole moments (11.63 D and 9.82 D), correlating with their diminished adhesive strengths (Figure 6f).

Overall, these results highlight the pivotal role of synergistic noncovalent interactions, particularly electrostatic interactions, dipole–dipole interactions, and hydrogen bonding, in dictating the adhesive performance of the dual‐end modular system. The absence of either terminal group substantially compromises adhesion on both hydrophilic (Steel) and hydrophobic (PET) substrates, with the phosphonium salt moiety—responsible for electrostatic and dipole–dipole interactions—exerting a more pronounced contribution than the carboxylic acid group, which primarily engages in dipole–dipole interactions and hydrogen bonding. Moreover, steric and electronic effects introduced by phosphonium substituents modulate molecular packing and supramolecular interactions, ultimately shaping the structural and mechanical behavior of the adhesive material. Notably, the higher adhesion strength of **A7** on PET compared to **A5** and **A6** further underscores the dominant role of the phosphonium salt moiety in driving interfacial interactions on hydrophobic substrates, relative to the contributions of carboxyl‐mediated dipole–dipole interactions and hydrogen bonding.

### 2.6 Accelerated Curing and Adhesion Enhancement via Deep Eutectic Solvent Integration

**Figure 7:**
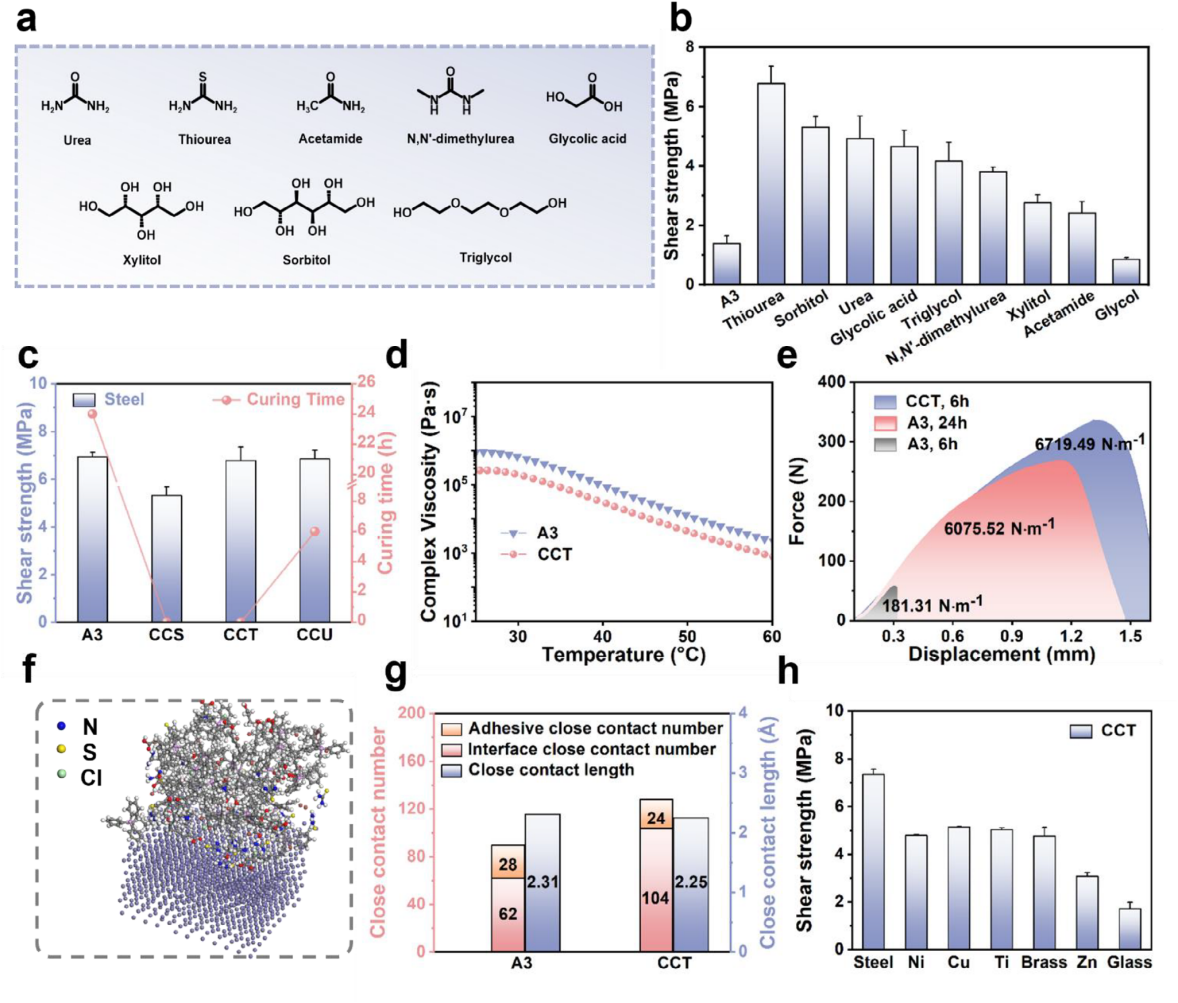
Accelerated curing and adhesion enhancement of **A3** via deep eutectic solvent integration. a) Molecular structures of the selected hydrogen bond donors (HBDs). b) Instantaneous adhesion strength of various HBD‐based DES formulations on Steel. c) Maximum adhesion strengths and corresponding curing times for **A3**, CCS, CCT, and CCU. d) Temperature‐dependent rheological profiles of CCT and **A3**. e) Force‐displacement curves in lap‐shear tests for CCT and **A3** at different curing times. f) Molecular dynamics simulation showing the lowest‐energy configuration of CCT on Fe at 25 °C. g) Comparison of the number and average length of close contacts between **A3** and CCT h) Lap‐shear adhesion strength of CCT on various hydrophilic substrates (curing time: 6 h). All data were presented as mean ± SD (n =3–5 independent samples).

The incorporation of deep eutectic solvents (DESs) has emerged as a promising strategy to enhance the performance of supramolecular adhesives.^[^ ^26^, ^29^ ^]^ Consequently, modular adhesive **A3** was combined with various DES formulations to bolster its adhesion capabilities. Screening of hydrogen bonding acceptors identified choline chloride (ChCl) as the optimal component (Figures S25 and S26, Supporting Information). When paired with a variety of hydrogen bonding donors (HBDs), most combinations led to instantaneous high adhesion, with the peak lap‐shear strength reaching up to 6.78 MPa (**Figure** **7**a,b). In contrast, unmodified adhesive **A3** exhibited only 1.38 MPa of immediate adhesion and required a full 24‐h curing period to reach optimal strength. Among the most effective formulations, **A3** + ChCl + Thiourea (CCT), **A3** + ChCl + Sorbitol (CCS), and **A3** + ChCl + Urea (CCU) were selected for further evaluation. CCT and CCS were able to achieve optimal adhesion instantaneously, whereas CCU required 4 h to reach similar performance (Figure 7c; Figure S27, Supporting Information). These results demonstrate that DES incorporation significantly accelerates curing, enhancing practical usability and expanding the application scope of the modular adhesive system. Time‐dependent rheological measurements provide direct experimental evidence that the DES‐containing formulation (CCT) promotes rapid supramolecular network formation during curing, as indicated by the swift stabilization of both storage modulus (G’) and viscosity. In contrast, the **A3**‐only system displayed a significantly slower curing process (Figure S31, Supporting Information).

Subsequent optimization of DES loading confirmed that **A3** remained the principal contributor to adhesion performance, while DESs served as performance enhancers (Figures S28 and S29, Supporting Information). Spectroscopic analysis revealed a shift in the C═O stretching band from 1713.0 to 1716.4 cm^−1^ upon DESs incorporation, indicating the formation of a new supramolecular network (Figure S30, Supporting Information). Rheological measurements further demonstrated that the CCT formulation exhibits lower viscosity than unmodified **A3** at elevated temperatures, improving substrate wettability and spreading efficiency, which in turn facilitates high initial adhesion strength (Figure 7d).

Mechanical characterization of debonding energy confirmed the dramatic impact of DESs on interfacial behavior. The CCT adhesive exhibited a debonding energy of 6719.49 N·m^−1^, comparable to **A3** after 24‐h curing (6075.52 N·m^−1^), yet over 37‐fold higher than that of **A3** at the 6‐h mark (181.31 N·m^−1^, Figure 7e). This underscores the exceptional rapid interfacial stabilization capacity imparted by DES integration. Molecular dynamics simulations supported these findings by revealing a substantial increase in the number of interfacial non‐covalent interactions (from 62 to 104) and a corresponding reduction in average interaction distances, both of which facilitate rapid adhesive‐substrate complexation (Figure 7f,g).

Practical lap‐shear testing across various metal substrates confirmed that DES‐modified formulations, particularly CCT, yield moderate but consistent strength enhancements. (Figure 7h). Thermal analysis via DSC showed a depressed T_g_ of −11.10 °C for the CCT formulation, compared to −3.15 °C for the pristine adhesive **A3** (Figure S32, Supporting Information). This decrease in T_g_ promotes greater molecular flexibility at lower temperatures, thereby enhancing conformational adaptability, improving interfacial wettability on rigid surfaces, and contributing to optimized stress distribution under thermal conditions.

## 3 Conclusion

We report a dual‐teminal modular design strategy to construct low‐molecular‐weight supramolecular adhesives that combine strong, reconfigurable, and recyclable adhesion. By integrating carboxylic acid and triphenylphosphonium terminals via a flexible alkyl linker, the adhesive forms a thermally responsive supermolecular network through synergistic hydrogen bonding, dipole–dipole interactions, and electrostatic interactions. This minimalist design affords high adhesive strength (up to 6.95 MPa), rapid curing, broad substrate compatibility (including PET and PTFE), and excellent stability across solvents, humidity, and extreme temperatures. Experimental and theoretical mechanistic investigation reveal that the terminal‐group synergy and high molecular dipole moments play decisive roles in governing cohesion and interfacial interactions. Moreover, incorporating DESs significantly accelerates curing and enhances toughness, offering a facile route for practical deployment. This work establishes a generalizable framework for designing programmable, sustainable adhesives via modular noncovalent assembly.

## Conflict of Interest

The authors declare no conflict of interest.

## Supporting information



Supporting Information

Supplemental Video 1

Supplemental Video 2

Supplemental Video 3

Supplemental Video 4

## Data Availability

The data that support the findings of this study are available in the supplementary material of this article.
