# Supplementary material for: Dual‐Terminal Molecular Strategy for Robust and Reversible Supramolecular Adhesion
Source: Adv Sci (Weinh). 2025 Aug 29;12(43):e11818. doi: 10.1002/advs.202511818 (PMC12631938; doi:10.1002/advs.202511818)
Supplement: Supplementary file 1 — Supporting Information [file ADVS-12-e11818-s002.docx]

**Supporting Information for**

**Dual-terminal molecular strategy for robust and reversible supramolecular adhesion**

Shiru Wang,^[a]^ Liang Meng,^[a]^ Feng Li,^[b]^ Yuru Wang,^[a]^ Yongri Liang,^[c]^ Guangming Lu,^[d]^ Keju Sun,^[a]^ Yingdan Liu^*[c]^ and Jingyue Yang^*[a]^

[a] State Key Laboratory of Metastable Materials Science and Technology, Nano-bio-technology Key Lab of Hebei Province, Applying Chemistry Key Lab of Hebei Province, Yanshan University, Qinhuangdao 066004, P. R. China.

[b] Key Laboratory of Green Chemistry & Technology of Ministry of Education, College of Chemistry, Sichuan University, 29 Wangjing Road, Chengdu, Sichuan, 610064, China.

[c] Center of Advanced Structural Materials, State Key Lab of Metastable Materials Science and Technology, School of Materials Science and Engineering, Yanshan University, No. 438 West Hebei Avenue, Qinhuangdao, Hebei, 066012, P.R. China.

[d] State Key Laboratory of Advanced Marine Materials, Ningbo Institute of Materials Technology and Engineering, Chinese Academy of Sciences, Ningbo, 315201, China.

*Corresponding author. Email: [yangjingyue@ysu.edu.cn](mailto:yangjingyue@ysu.edu.cn); ydliu@ysu.edu.cn*

**This PDF file includes the following:**

**Schemes S1-4 (Page S4-7)**

**Tables S1-4 (Page S16-17, S21, S25)**

**Figures S1-32 (Pages S8–24)**

**Supplementary References (Page S26)**

**Supporting Information Videos 1-4**

***Materials and methods***

All reagents are commercially available and can be used without further purification. 6- bromodecanoic acid, 8- bromodecanoic acid, 10- bromodecanoic acid, 11- bromodecanoic acid and triphenyl phosphorus were purchased from Shanghai Bide Pharmaceutical Technology Co., Ltd. (China, Shanghai). Thiourea and choline chloride were purchased from Shanghai Shuangde Pharmaceutical Technology Co., Ltd. (China, Shanghai). Dichloromethane was purchased from Beijing inoke Technology Co., Ltd. (Beijing, China). Anhydrous acetonitrile was purchased from Aladdin Reagent Co., Ltd. (China). Toluene was purchased from Qinhuangdao Zhisheng Trading Co., Ltd. (China).

The ^1^H NMR and the ^13^C spectrum were recorded on a 400 MHz Bruker Advance III spectrometer at room temperature using CDCl_3_ as solvent. Fourier transform infrared spectrometer (FT-IR) (NICOLET iS10) was used to analyze the samples before and after adhesive formation. Scanning electron microscopy (SEM) was performed on a high-resolution Regulus SU8230 scanning electron microscope at an accelerating voltage of 20 kV. Before the test, the surfaces of the samples were deposed by gold spraying for 90 seconds. Thermogravimetric analysis (TGA) was carried out using a Mettler Toledo TGA/DSC 3^+^ instrument with temperatures ranging from room temperature to 800 °C at a heating rate of 10 °C/ min^−1^ in a nitrogen atmosphere. Transition temperatures of the materials were determined on a TA Instruments 250 differential scanning calorimeter (DSC) under a nitrogen atmosphere. The test sample (∼5 mg) was heated from −80 °C to 200 °C with a rate of 10 °C min^−1^. Rheology measurements were performed on an Anton Paar MCR 502. The laminator model PP15-E with a diameter of 15 mm and a gap of 1 mm was chosen. The lap-shear adhesion strength was investigated by using a DY-LDW-5000N universal tensile testing machine.

FT-IR spectra were recorded using a Bruker INVENIO S spectrometer equipped with a DTGS detector. A Harrick in situ transmission cell was used for temperature control. The sample was heated from 30°C to 150°C and subsequently cooled back to 30°C, with spectra collected at 10°C intervals. Each temperature was maintained for 5 minutes before data acquisition to ensure thermal equilibration. Two-dimensional correlation spectroscopy (2DCOS) The temperature-dependent FT-IR spectra of **A3** from 30 to 150 °C were used for analysis.

In the contour maps, red colors are defined as positive intensities, and blue colors are defined as negatives.

The responsive order of different groups can be judged by Noda’srule. The molecular geometrical structures were optimized by density functional theory (DFT) calculation using Gaussian program package at the B3LYP/6-31G^[1]^ level and then B3LYP/6-311G (d, p) level. The interaction energies between two molecules were calculated by the following formula:

${\triangle E}_{\mathrm{interaction}}={\triangle E}_{\mathrm{total}}-{\triangle E}_{\mathrm{molecule}\left( 1 \right)}-{\triangle E}_{\mathrm{molecule}\left( 2 \right)}$ （1）

The work of debonding was calculated as follows:

$Work of debonding=\frac{\int_{0}^{x} Fdx}{A_{overlap area}}$ (2)

Where $\int_{0}^{x} Fdx$ is the measured overlap area of the adhesion joint.

***Molecular dynamics simulation***

Molecular dynamics simulations of the modular adhesive **A3** on Steel and PET were performed using Materials Studio to investigate the adhesion mechanisms on hydrophilic and hydrophobic substrates. The sizes of the adhesive and the substrate in the established two-layer model were 3.0 × 3.0 × 2.0 nm^3^ and 3.0 × 3.0 × 1.5 nm^3^, respectively. The Smart algorithm under the COMPASSII is used for geometry optimization to obtain the global minimum energy configuration. Then, 6 heating ramps per cycle with a total of 5 cycles of annealing from 300 K to 600 K were performed under the constant volume and constant temperature (NVT) ensemble. The configuration corresponding to minimum energy was extracted for further dynamics simulation. Dynamics simulations under the NVT ensemble were conducted for 200 ps with a time step of 1 fs at different temperatures. The Ewald method and atom-based method were employed to calculate the Coulomb interactions and van der Waals interactions between adhesives and substrates, respectively. The Nosé-Hoover thermostat was utilized to regulate and control the temperature throughout the simulation process. Finally, the interaction energy between the adhesive and the substrate at different temperatures was calculated by the following equation, and a script was used to count the number and length of the close contacts in the adhesive or two-layer model systems at 298 K.

$E_{\mathrm{interfacial}}=E_{\mathrm{total}}-(E_{\mathrm{adhesive}}+E_{\mathrm{substrate}}$) (3)

Where E_interfacial_ is the interaction energy between the adhesive and the substrates; E_total_ is the total potential energy of the adhesive and substrates; E_adhesive_ and E_substrate_ represent the potential energy of the adhesive and substrates (Fe or PET).

*Synthesis of compound* ***A3***

**Scheme S1.** Synthetic route of compounds **A3**.

Taking the synthesis of **A3** as an example, the synthesis was carried out according to the literature^[2]^. 10- bromodecanoic acid (10 g, 40 mmol) and triphenylphosphorus (10.49 g, 40 mmol) were added into a two-necked bottle, and 50 mL of toluene was added under argon protection, and the mixture was heated at 120 °C under reflux for 72 hours, during which a yellow jelly formed. Cooling the reaction product to room temperature, separating the solvent from the yellow colloid, then transferring the yellow colloid to a round-bottom flask, pulping with toluene until the product completely turns into light yellow powder, filtering, and repeating the above operations for three times. The compound was light yellow powder with a yield of 73%. The synthetic steps of **A1**, **A2** and **A4** are consistent with those of **A3**.

The ^1^H NMR spectrum of **A3** is shown in Figure S1:^.1^H NMR (400 MHz, Chloroform-*d*) δ 7.82 – 7.64 (m, 15H), 3.64 – 3.52 (m, 2H), 2.30 (t, *J* = 7.32 Hz, 2H), 1.62-1.46 (m, 6H), 1.25 – 1.1 (m, 8H).

The ^13^C NMR spectrum of **A3** is shown in Figure S2: ^13^C NMR (101 MHz, Chloroform-d) δ 177.20, 135.28, 135.25, 133.75, 133.65, 130.77, 130.65, 118.74, 117.89, 77.68, 77.36, 77.04, 34.66, 30.36, 30.20, 28.85, 28.78, 24.80, 22.58, 22.54, 22.49.

HRESIMS of **A3**: m/z calcd for C_28_H_34_BrO_2_P, 433.2291, [M - Br] ^+^; found 433.2310 [M - Br] ^+^.

The ^1^H NMR spectrum of **A1** is shown in Figure S3: ^1^H NMR (400 MHz, Chloroform-*d*) δ 7.81 – 7.63 (m, 15H), 3.56 (app t, *J* = 12.52 Hz 2H), 2.29 (app t, *J* = 6.79 Hz, 2H),1.65 – 1.57 (m, 6H)^[3]^.

The ^1^H NMR spectrum of **A2** is shown in Figure S4: ^1^H NMR (400 MHz, Chloroform-d) δ 7.79 – 7.26 (m, 15H), 3.59 – 3.51 (t, *J* = 13.28 Hz, 2H), 2. 30-2.26(t, *J* = 7.32 Hz, 2H), 1.60 – 1.46 (m, 6H), 1.30-1.18 (m, 4H).

The ^13^C NMR spectrum of **A2** is shown in Figure S5: ^13^C NMR (101 MHz, Chloroform-d) δ 176.85, 135.14, 135.11, 133.58, 133.48, 130.63, 130.50, 118.54, 117.68, 77.48, 77.16, 76.84, 34.49, 29.97, 29.82, 28.25, 28.13, 24.53, 22.28, 22.24.

The ^1^H NMR spectrum of **A4** is shown in Figure S6: ^1^H NMR (400 MHz, Chloroform-d) δ 7.78 – 7.71 (m, 9H), 7.65 (m, 6H), 3.61 – 3.49 (m, 2H), 2.28 (t, *J* = 7.36 Hz, 2H), 1.53 (m, 6H), 1.21 – 1.10 (m, 10H)^[4]^.

*Synthesis of compound* ***A5****:*

10- bromodecanoic acid (1 g, 4 mmol), tricyclohexylphosphine (1.12 g, 4 mmol) and potassium iodide (26.55 mg, 0.155 mmol) were added to the round bottom flask in turn. Subsequently, 10 mL of DCM was added at room temperature to completely dissolve it and start the reaction. The reaction stopped after 24 hours. The reaction mixture was purified by silica gel column chromatography using 40% EA/PE as eluent, and finally concentrated to obtain the product. The obtained compound **A5** is a light yellow transparent viscous liquid with a yield of 19%.

**Scheme S2.** Synthetic route of compounds **A5**.

The ^1^H NMR spectrum of **A5** is shown in Figure S7: ^1^H NMR (400 MHz, Chloroform-*d*) δ 2.48 (app q, 3H), 2.31 – 2.19 (m, 4H), 1.86 (dd, *J* = 29.5, 8.4 Hz, 12H), 1.69 (d, *J* = 12.68 Hz, 3H), 1.42 (m, 18H), 1.20 (app s, 11H).

The ^13^C NMR spectrum of **A5** is shown in Figure S8: ^13^C NMR (101 MHz, Chloroform-*d*) δ 77.48, 77.16, 76.84, 35.76, 35.15, 31.69, 31.54, 31.26, 31.14, 27.76, 27.66, 27.06, 26.94, 26.60, 26.43, 26.40, 26.23, 26.21.

HRESIMS of **A5**: m/z calcd for C_28_H_52_O_2_PBr, 451.3699, [M - Br] ^+^; found 451.3702[M - Br] ^+^.

*Synthesis of compound* ***A6***

Add 10- bromodecanoic acid (1 g, 4 mmol) and Tris (4-methoxyphenyl) phosphine (1.55 g, 4.4 mmol) into a two-necked bottle, add 10 mL of acetonitrile under the protection of argon, and heat and reflux for 24 hours, and a brown gel appears. The reaction solution was cooled to room temperature, the solvent was removed by rotary evaporation, purified on silica gel column with 5% MeOH/DCM eluent, and the product was concentrated. Compound **A6** was obtained, which was brownish yellow and transparent, with a yield of 68%.

**Scheme S3.** Synthetic route of compounds **A6**.

The ^1^H NMR spectrum of **A6** is shown in Figure S9: ^1^H NMR (400 MHz, Chloroform-*d*) δ 7.64- 7.58 (m, 6H), 7.26-7.14 (m, 6H), 3.89 (s, 9H), 3.28 (d, *J* = 13.0 Hz, 2H), 2.30 (t, *J* = 7.3 Hz, 2H), 1.59 – 1.47 (m, 6H), 1.26 – 1.16 (m, 8H).

The ^13^C NMR spectrum of **A6** is shown in Figure S10: ^13^C NMR (101 MHz, Chloroform-*d*) δ 164.72, 164.69, 135.50, 135.39, 116.38, 116.24, 109.53, 108.60, 77.48, 77.16, 76.84, 56.10, 34.69, 28.67, 28.64, 24.79, 24.79, 23.47, 23.47, 22.51, 22.47.

HRESIMS of **A6**: m/z calcd for C_31_H_40_O_5_PBr, 523.2608, [M - Br] ^+^; found 523.2601 [M - Br] ^+^.

*Synthesis of compound* ***A7****:*

Under an argon atmosphere, triphenylphosphine (652.60 mg, 2.49 mmol) was placed in a two-necked flask, followed by the addition of decanoic acid (500 mg, 2.26 mmol) and toluene (4 mL). The reaction mixture was stirred under reflux for 24 h. After cooling to room temperature, a white solid precipitated, which was collected by filtration and recrystallized from ethyl acetate. The final product was obtained as a white powder in 55% yield.

**Scheme S4.** Synthetic route of compounds **A7**.

The ^1^H NMR spectrum of **A7** is shown in Figure S11: ^1^H NMR (400 MHz, Chloroform-*d*) δ 7.81 – 7.60 (m, 15H), 3.69 – 3.54 (m, 2H), 1.54 (q, *J* = 3.7 Hz, 4H), 1.14 (m, 12H), 0.76 (t, *J* = 7.12 Hz, 3H).

The ^13^C NMR spectrum of **A7** is shown in Figure S12: ^13^C NMR (101 MHz, CDCl_3_) δ 135.03 135.00, 133.61, 133.51, 130.55 130.42, 118.64, 117.79, 77.48, 77.16, 76.84, 31.71, 30.43, 30.28, 29.35, 29.11, 29.09, 23.02, 22.57, 22.53, 14.01.

HRESIMS of **A7**: m/z calcd for C_28_H_36_BrP, 403.2549, [M - Br] ^+^; found 403.2545[M - Br] ^+^.^
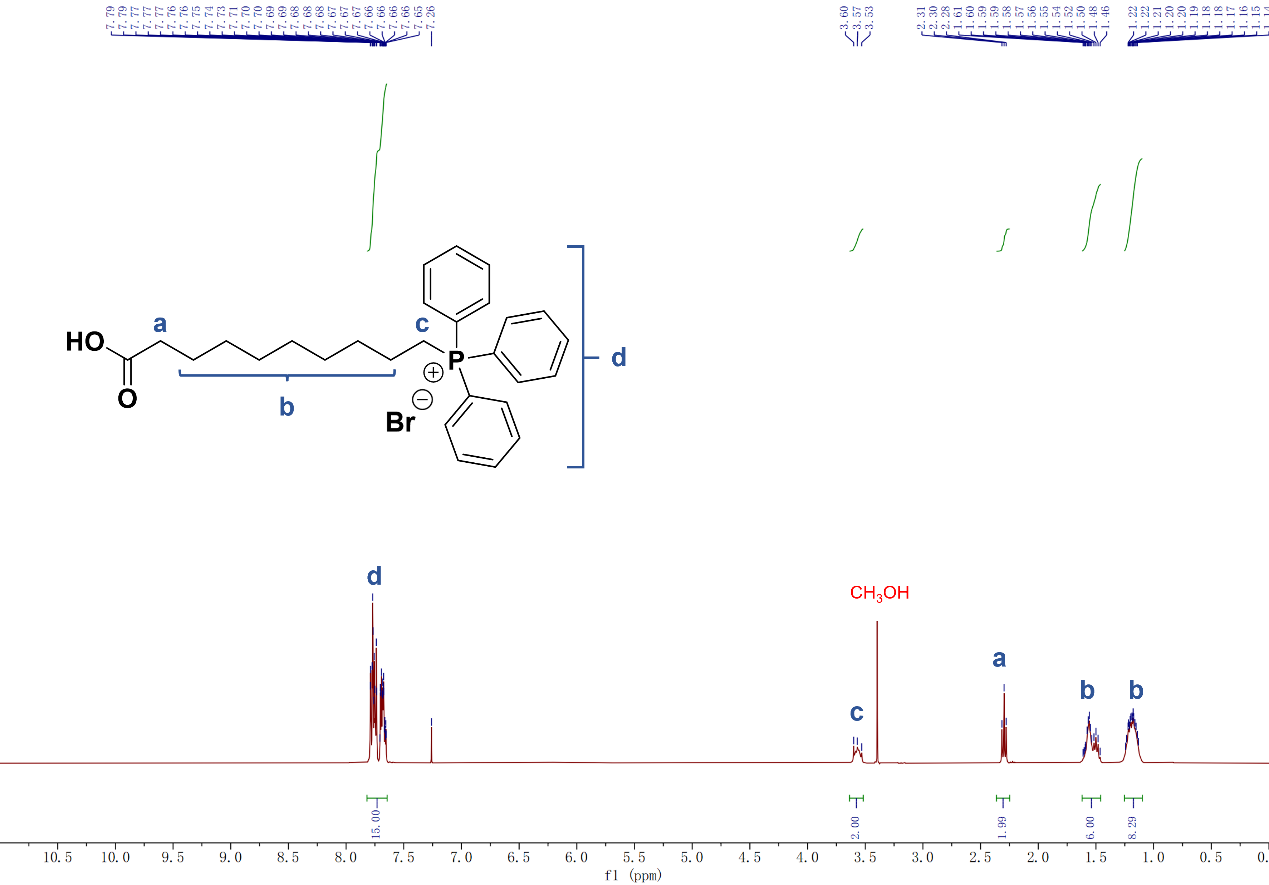
^

**Figure S1.** ^1^H NMR spectrum of **A3** in Chloroform-*d.*


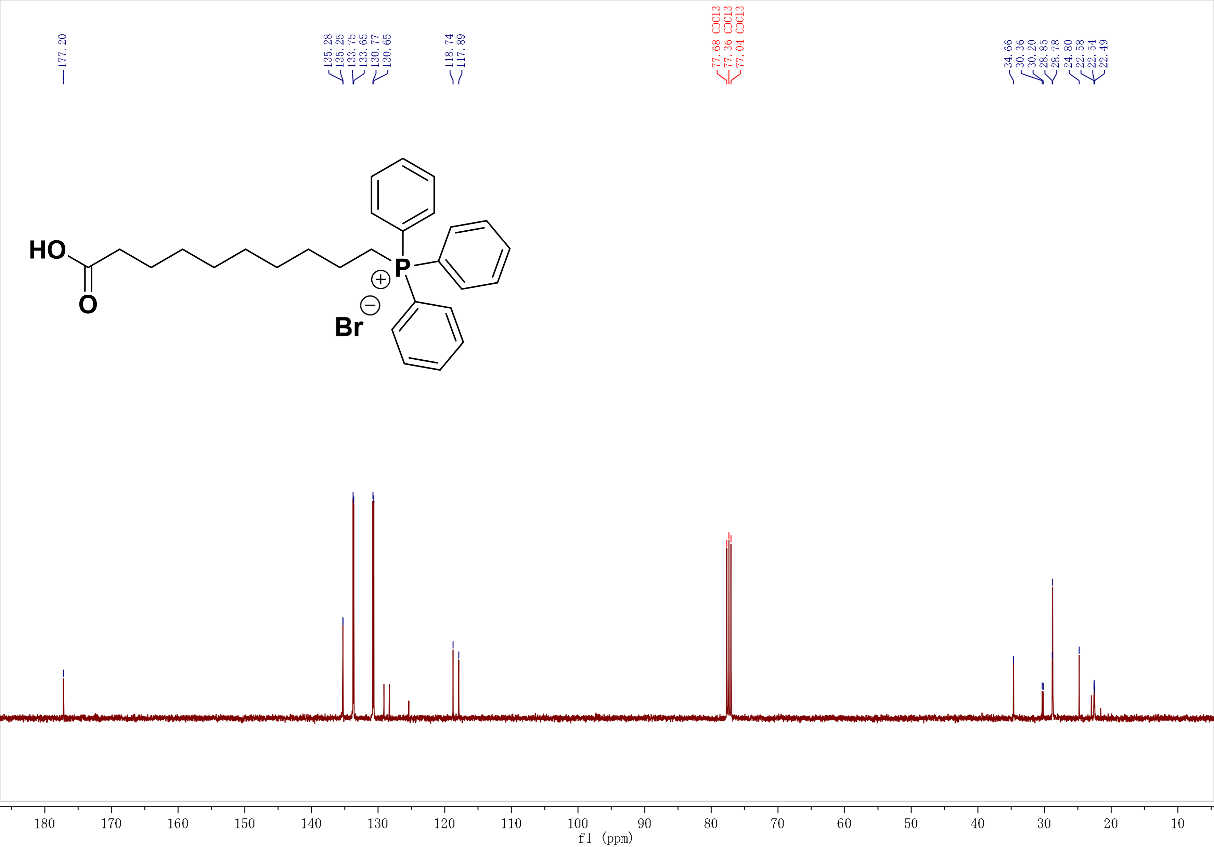


**Figure S2.** ^13^C NMR spectrum of **A3** in Chloroform-*d.*

^
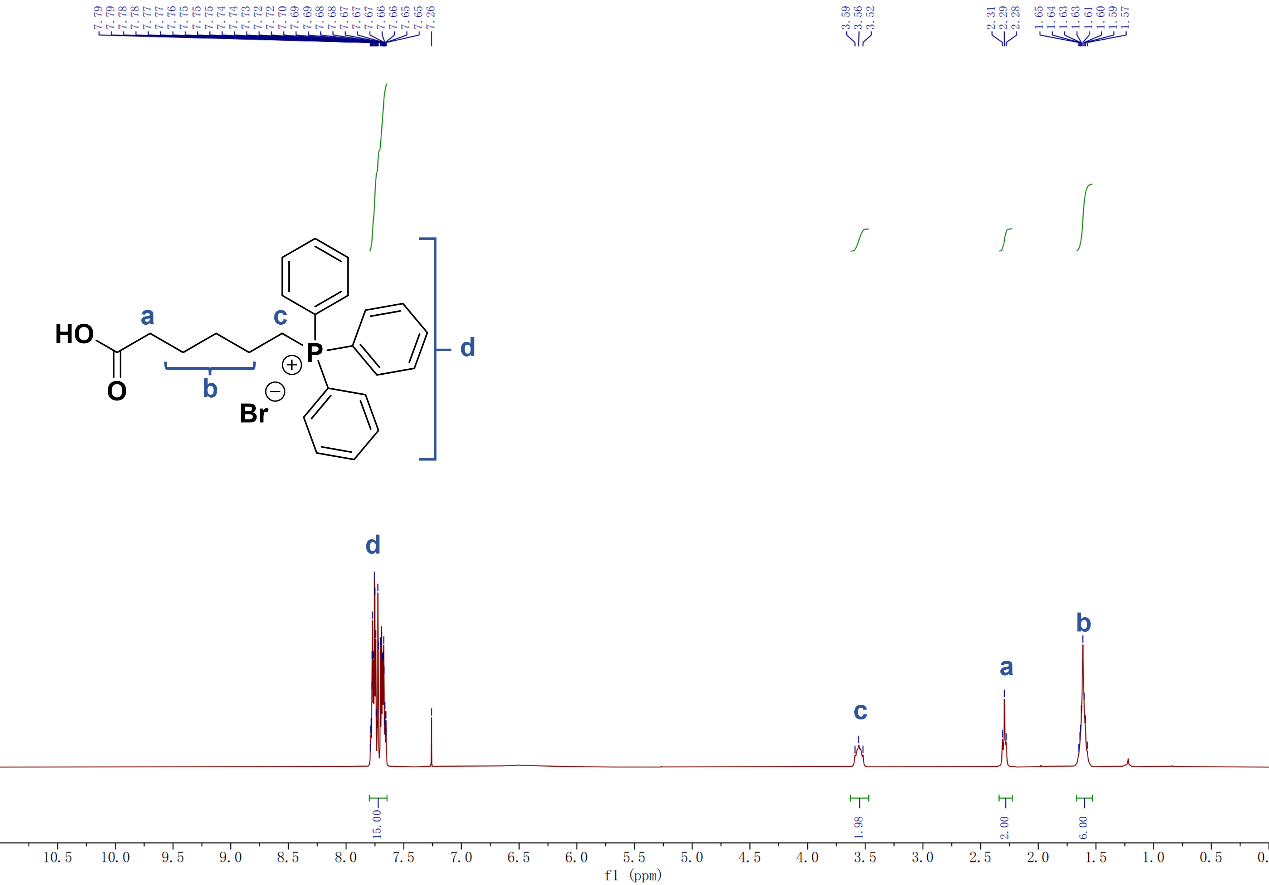
^

**Figure S3.** ^1^H NMR spectrum of **A1** in Chloroform-*d.*


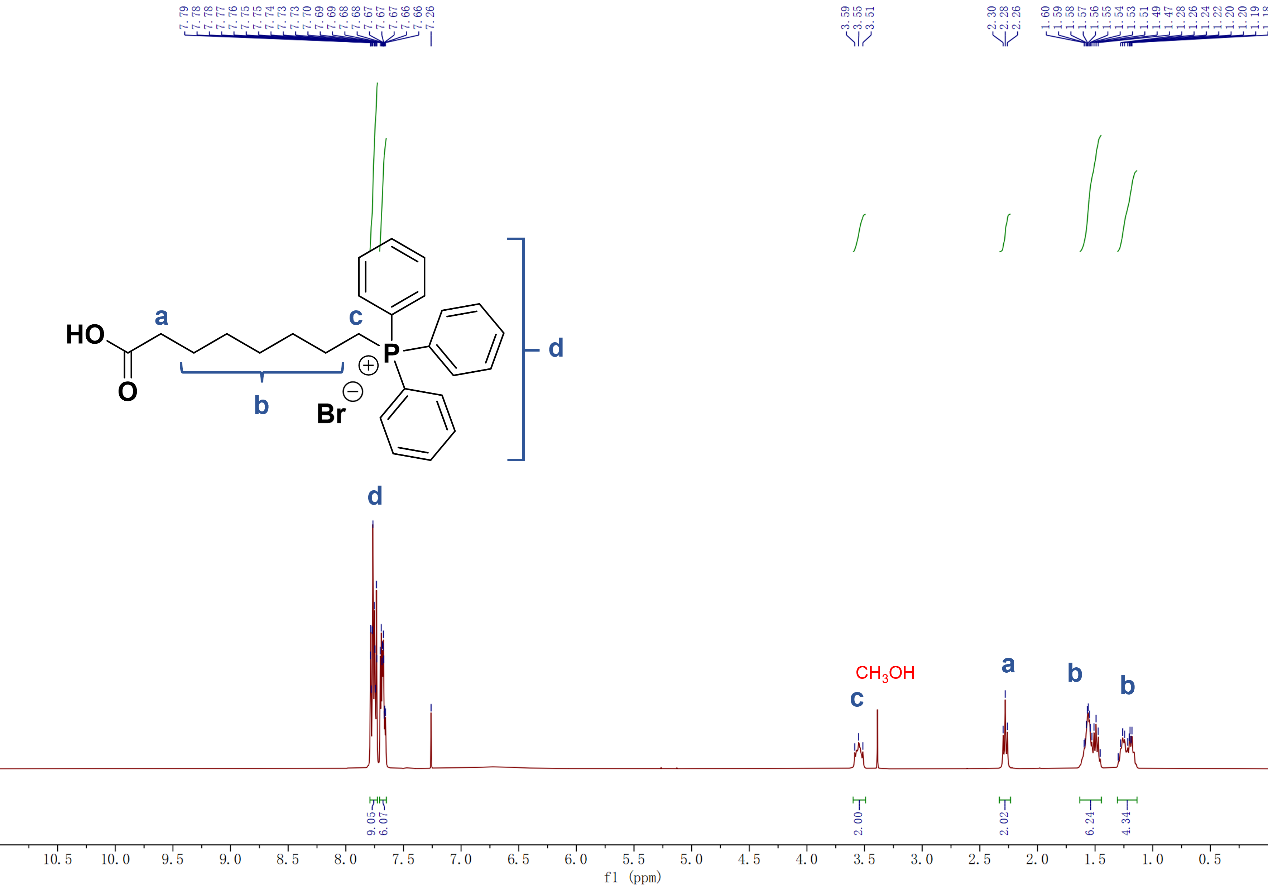


**Figure S4.** ^1^H NMR spectrum of **A2** in Chloroform-*d.*


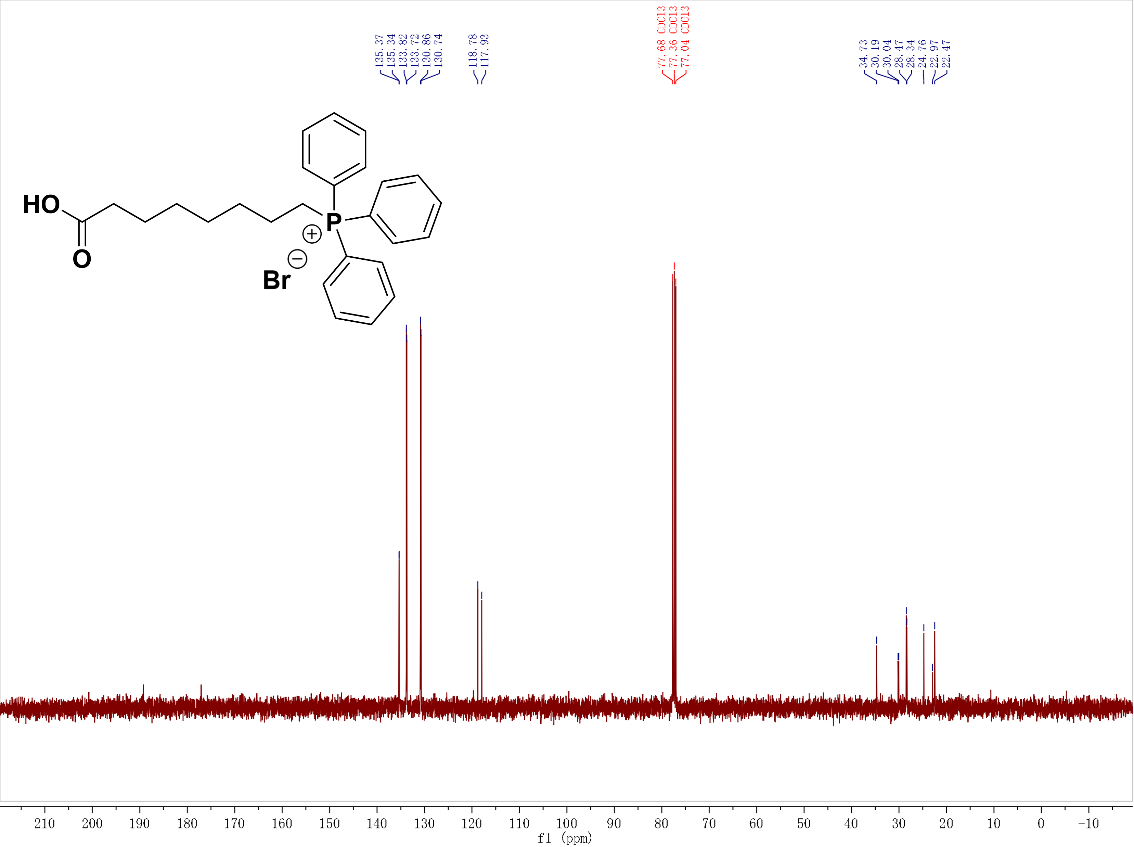


**Figure S5.** ^13^C NMR spectrum of **A2** in Chloroform-*d.*


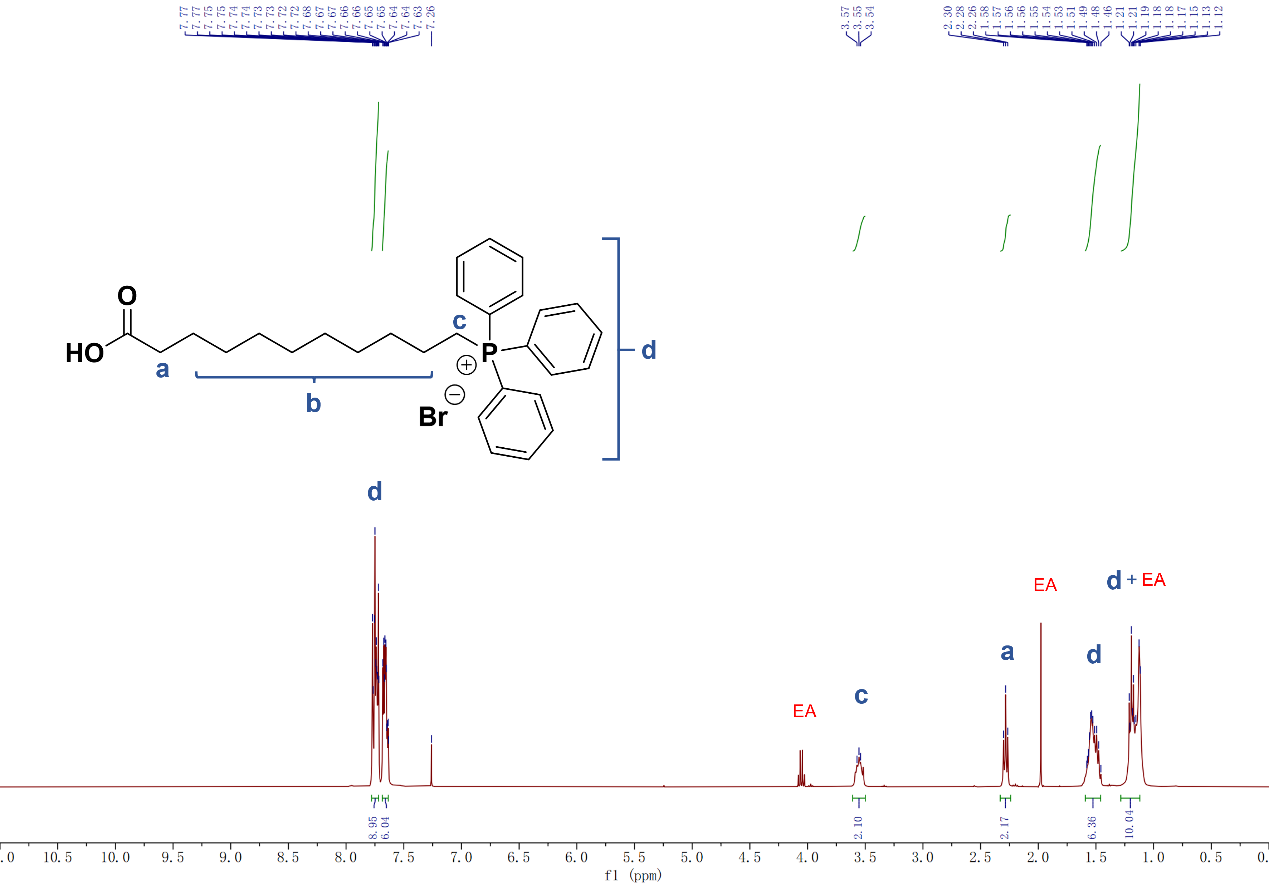


**Figure S6.** ^1^H NMR spectrum of **A4** in Chloroform-*d*.


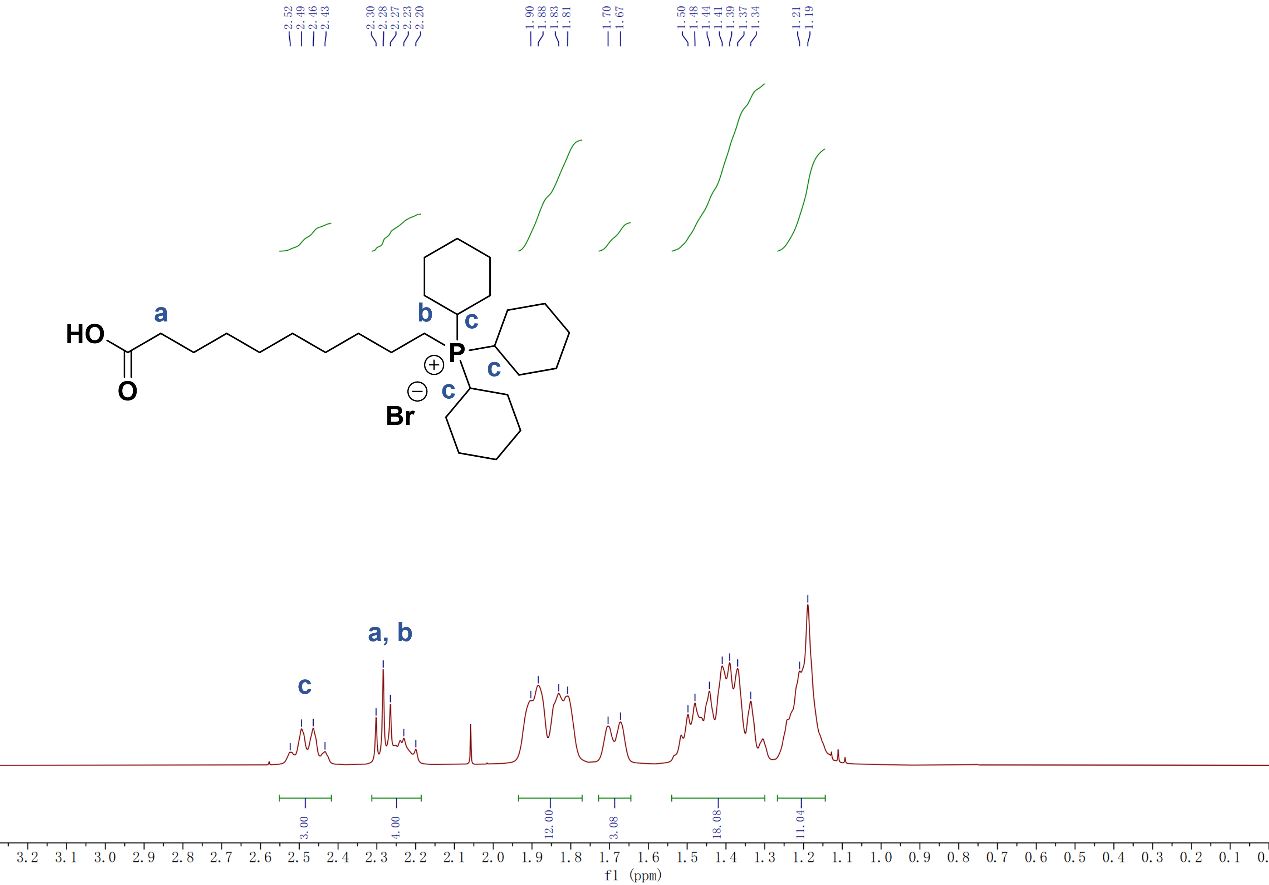


**Figure S7.** ^1^H NMR spectrum of **A5** in Chloroform-*d.*


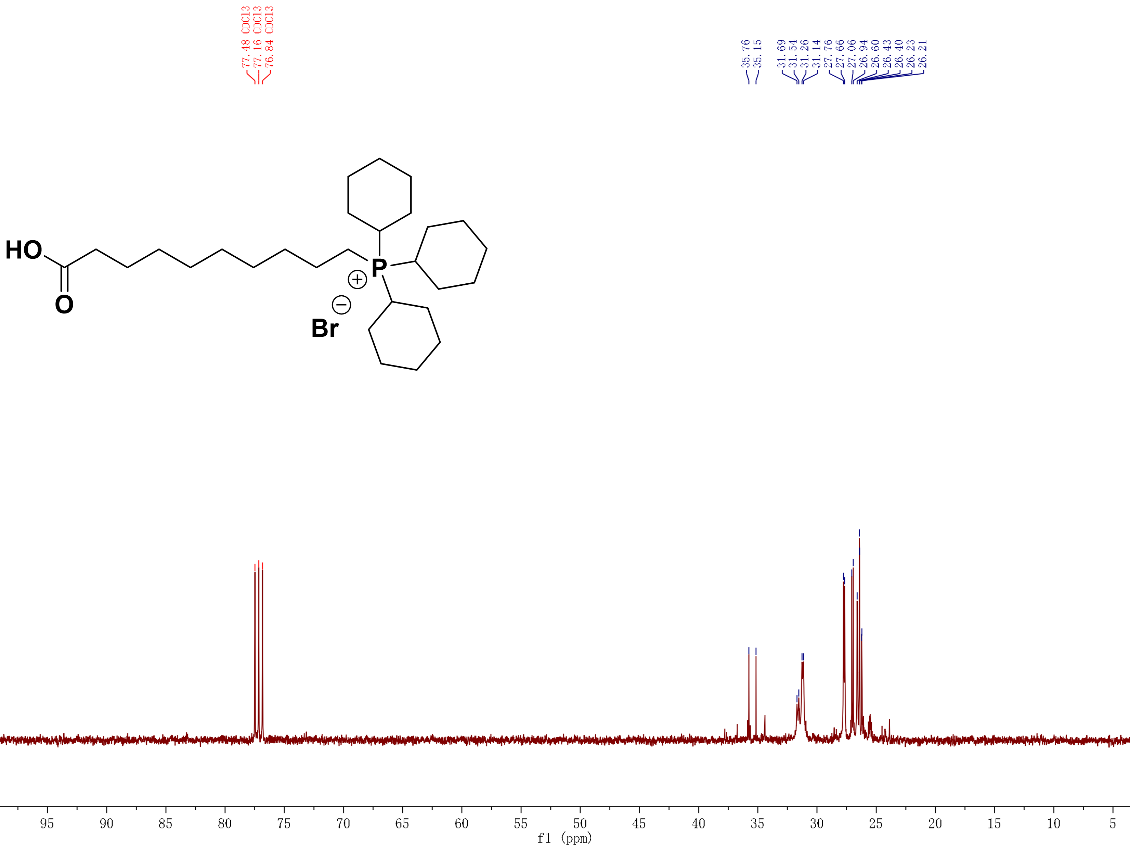


**Figure S8.** ^13^C NMR spectrum of **A5** in Chloroform-*d.*


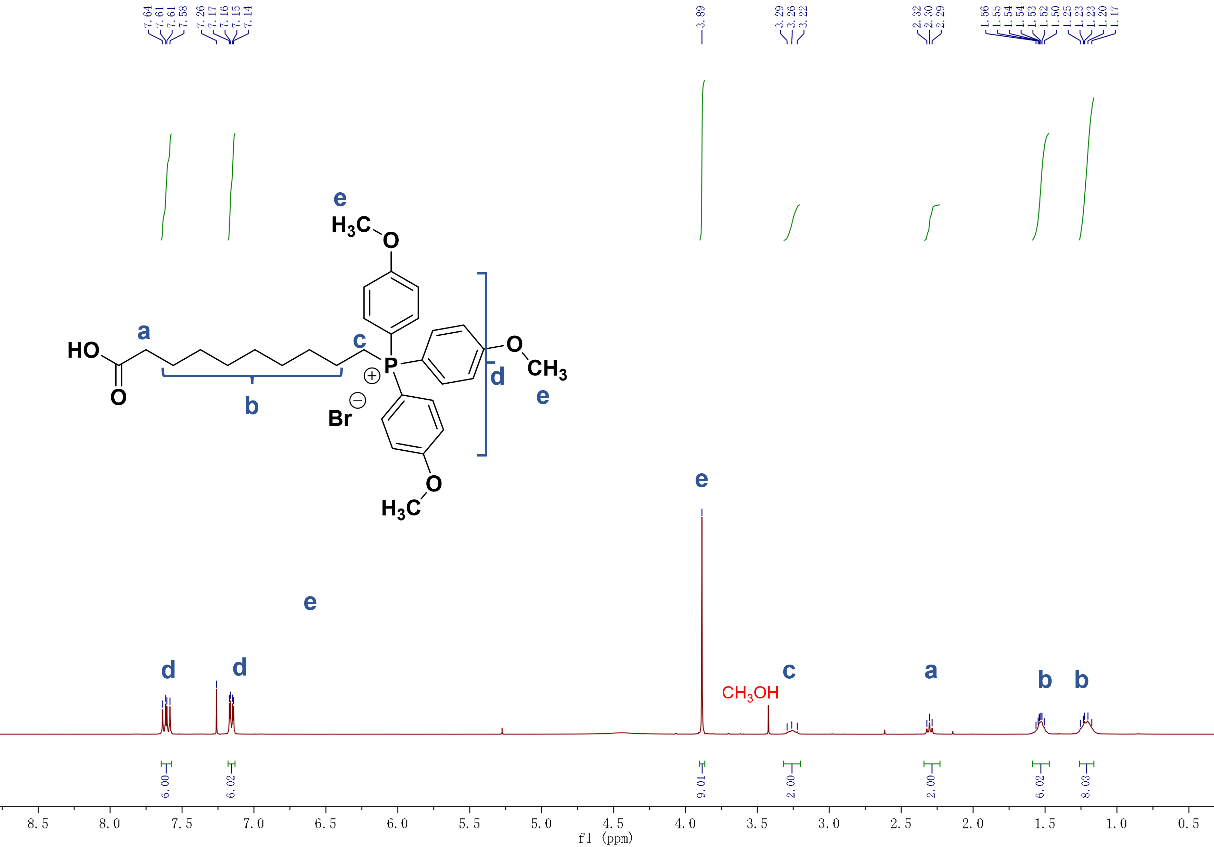


**Figure S9.** ^1^H NMR spectrum of **A6** in Chloroform-*d.*


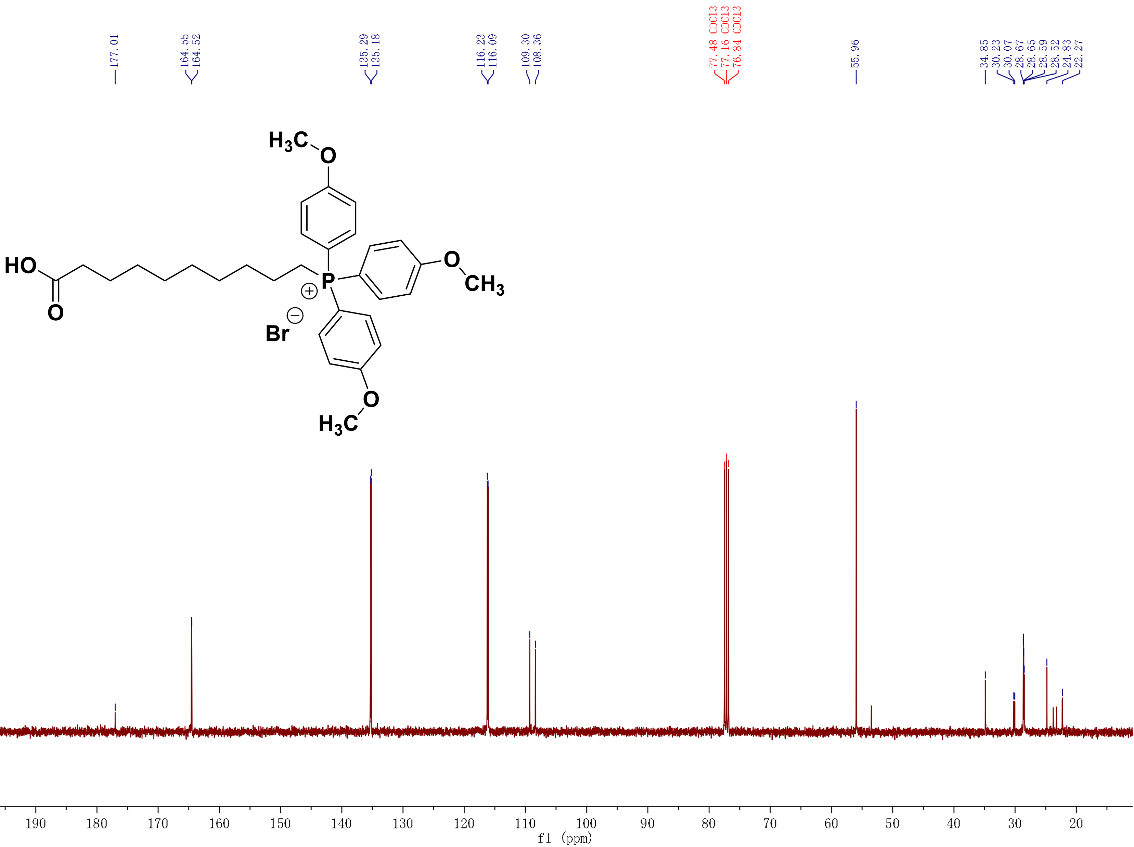


**Figure S10.** ^13^C NMR spectrum of **A6** in Chloroform-*d.*


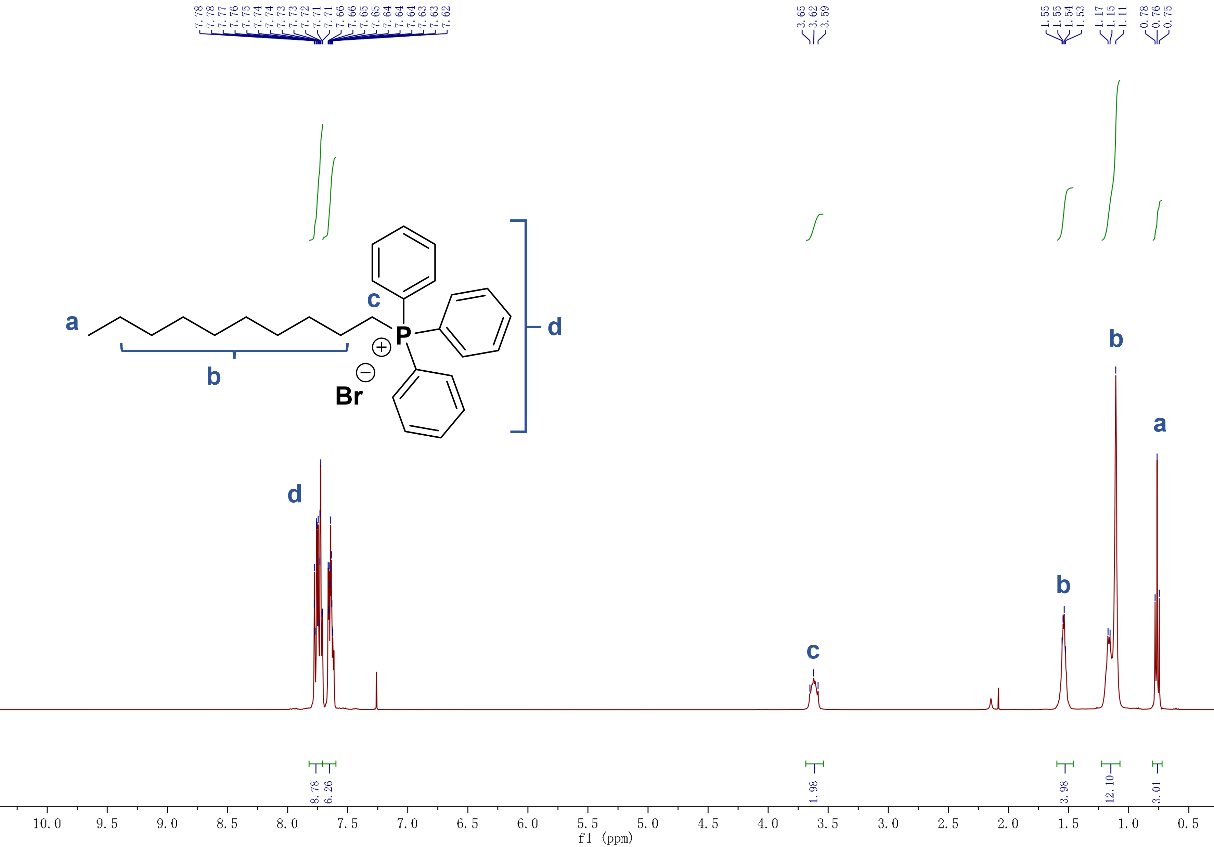


**Figure S11.** ^1^H NMR spectrum of **A7** in Chloroform-*d.*


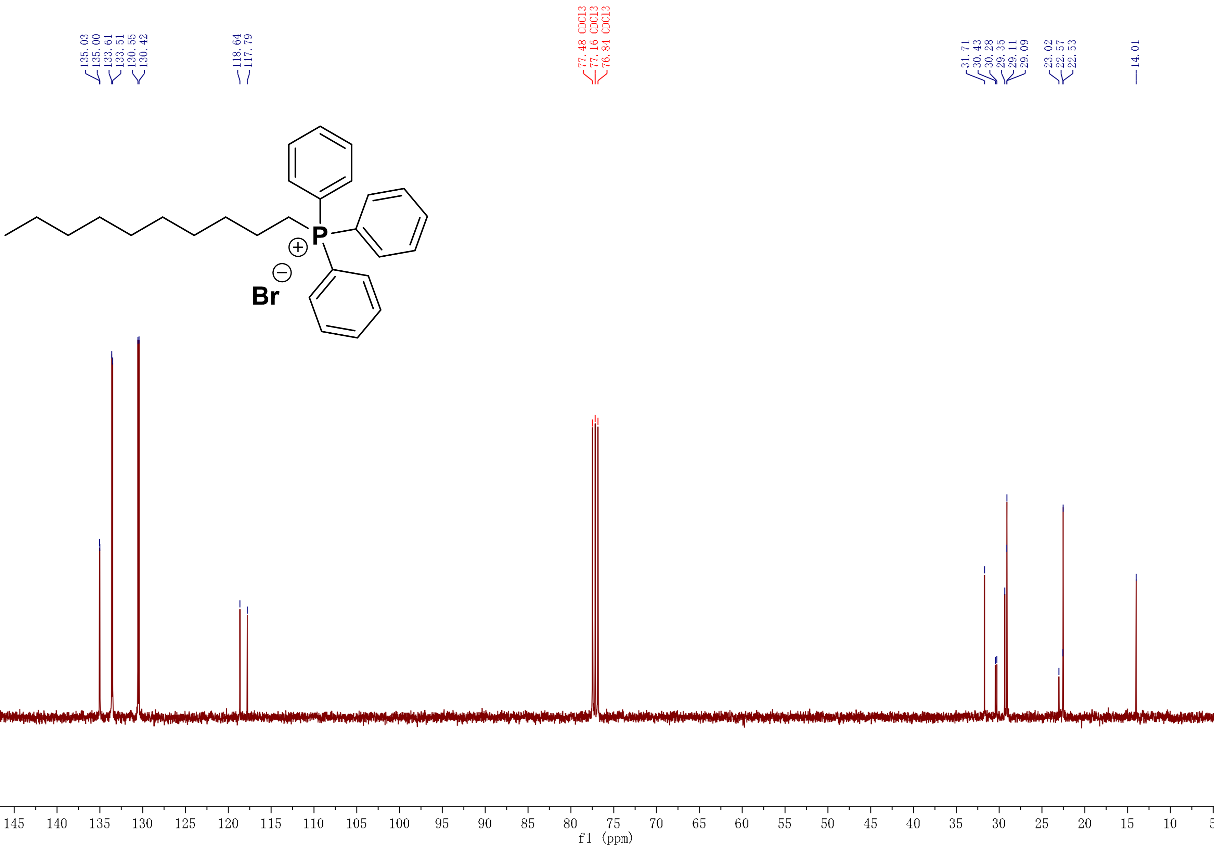


**Figure S12.** ^13^C NMR spectrum of **A7** in Chloroform-*d.*


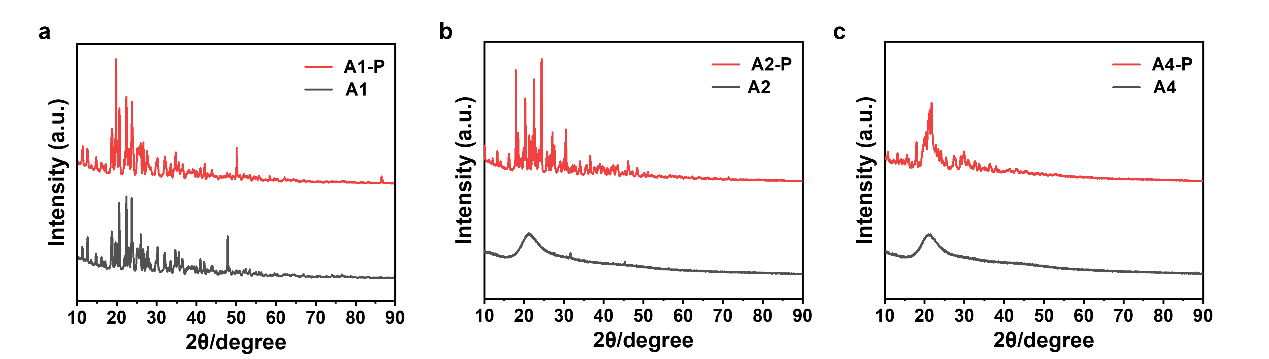


**Figure S13.** XRD diffractograms of **A1** (a)、**A2** (b) and **A4** (d).


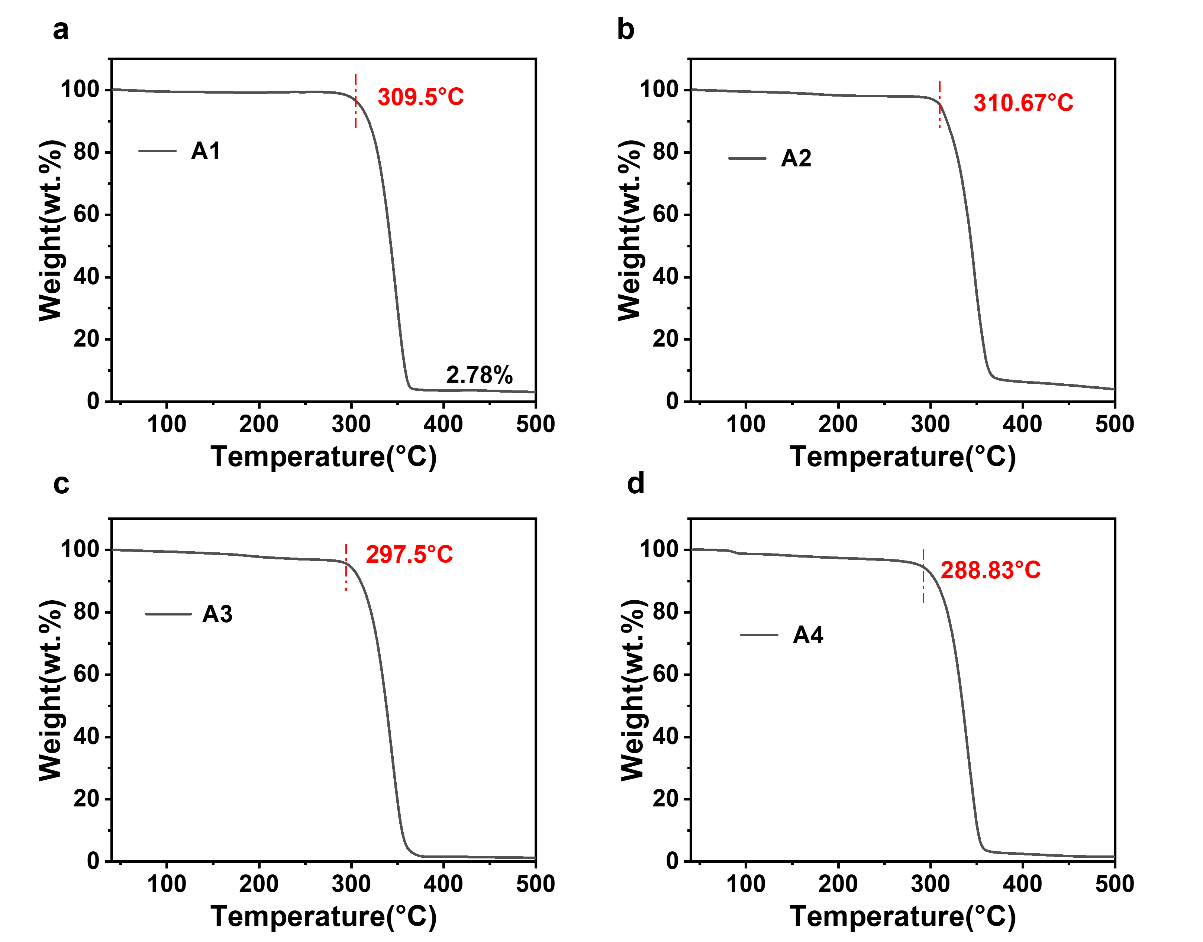


**Figure S14.** TGA curves of **A1** (a)、**A2** (b)、**A3** (c) and **A4** (d).

**Figure S15.** Frequency-dependent rheological tests of modular adhesive **A3**.

**Figure S16.** Work of debonding of **A2**、**A3** and **A4** on Steel substrate.

**Figure S17.** Time-dependent adhesion strengths of modular adhesive **A3** (RH = 20%, 25 °C).

**Figure S18.** Lap-shear bond strength of modular adhesive **A3** cured for 24 h in different humidity environments (“*” indicates substrate deformation).

| **Journals** | **Material** | **substrates** | **Adhesive Strength (Adhesion strength in air at RT)** |
| --- | --- | --- | --- |
| Macromol.Mater.Eng.**2022**,*307*,2200531 | catechol-decorated cationic adhesive polymer(CCAP) | PE  PI | 0.31 MPa  NA |
| Chem. Eng. J. **2022**, *442*, 136206. | LA-TA-FeCl_3_  (LTFE) | PE  PI | ≈20kPa  NA |
| Chem. Sci. **2023**, *14*, 3938–3948. | nucleobase-containing polyelectrolyte P_70-15-15_-C_8_ | PE  PI | 0.18 MPa  NA |
| Angew. Chem. Int. Ed. **2024**, *63*, e202403220. | IL-based molecular solids(P2) | PE  PI | 0.23 MPa  NA |
| **Our work** | **A3** | **PE**  **PI** | **1.27 MPa**  **3.16 MPa** |

**Table S1.** Comparison for adhesion performance with previously reported adhesives on PE and PI substrate (PI rarely reported).


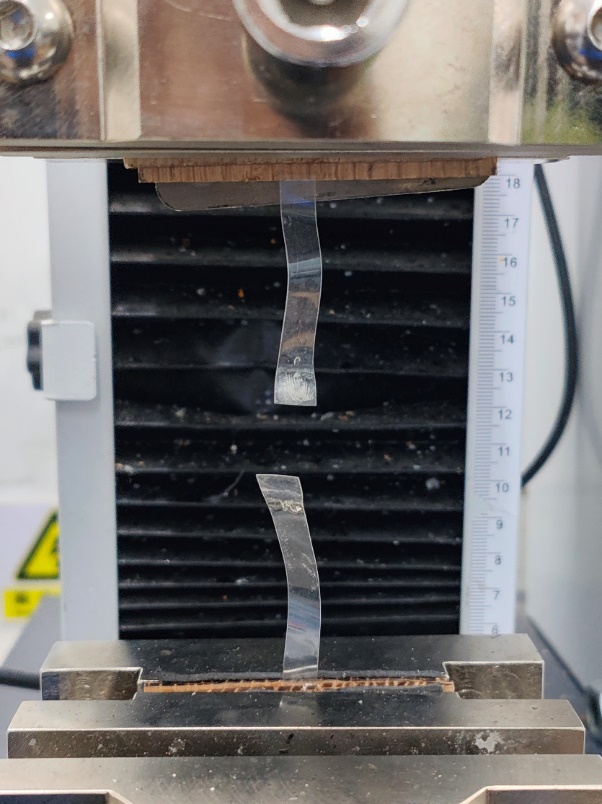


**Figure S19.** Deformed PET substrate in lap shear test

| **T（℃）** | **E_total_**  **（kcal/mol）** | **E_adhesive_**  **（kcal/mol）** | **E_Fe_**  **（kcal/mol）** | **E_interfacial_**  **（kcal/mol）** |
| --- | --- | --- | --- | --- |
| 25 | -161572 | -4110 | -155125 | -2337 |
| -18 | -161823 | -4312 | -155267 | -2244 |
| -80 | -162287 | -4604 | -155368 | -2315 |

**Table S2.** Interfacial adhesion energy (IAE) between Fe and **A3** adhesive under 25 °C、-18 ℃、-80 ℃ were respectively calculated by MD simulations.


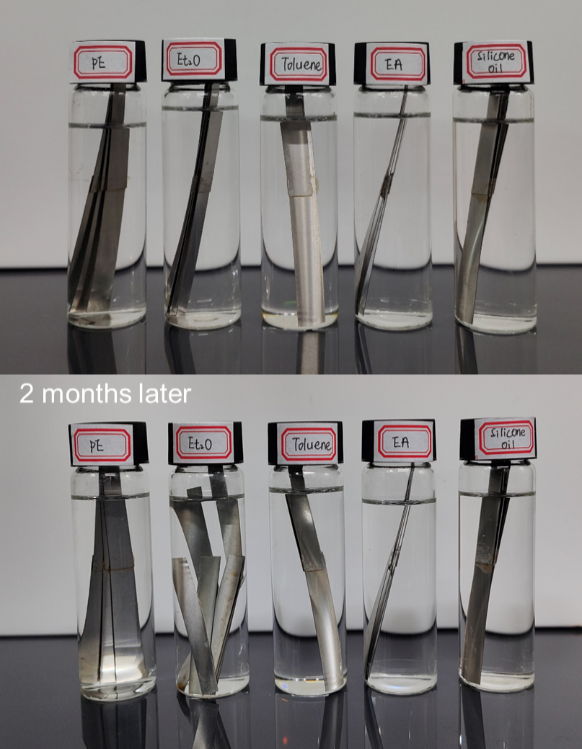

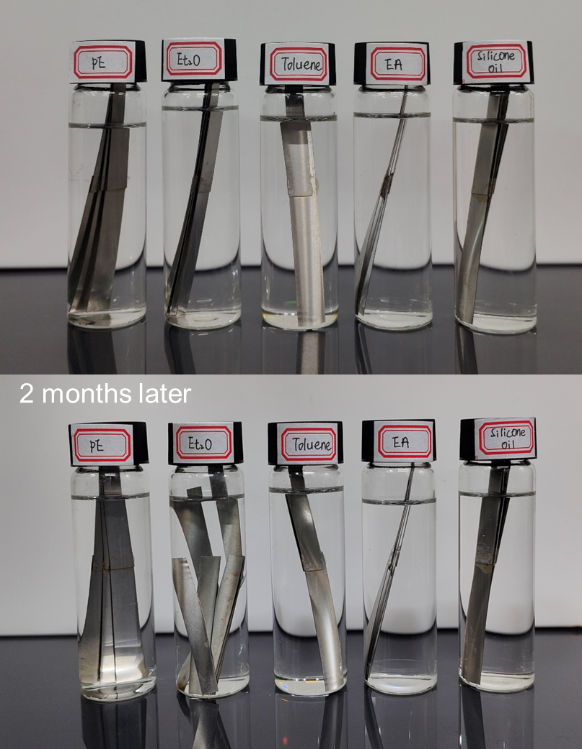


**Figure S20.** Digital image of **A3** bonded Steel sheets solvent immersion for 0 h (up) and after 2 months solvent immersion (down) in different solvents.


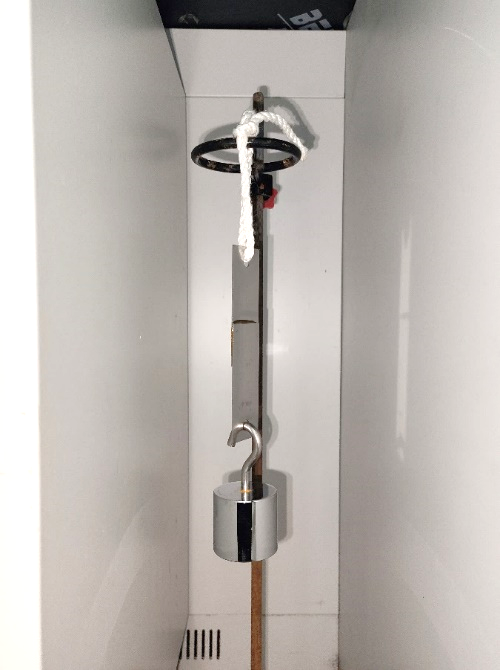


**Figure S21** Room temperature stability macroscopic testing of modular adhesive **A3** (Steel, 9 cm^2^).

**Figure S22.** TGA comparison of the modular adhesive **A3** in its initial state and after six recycling cycles.


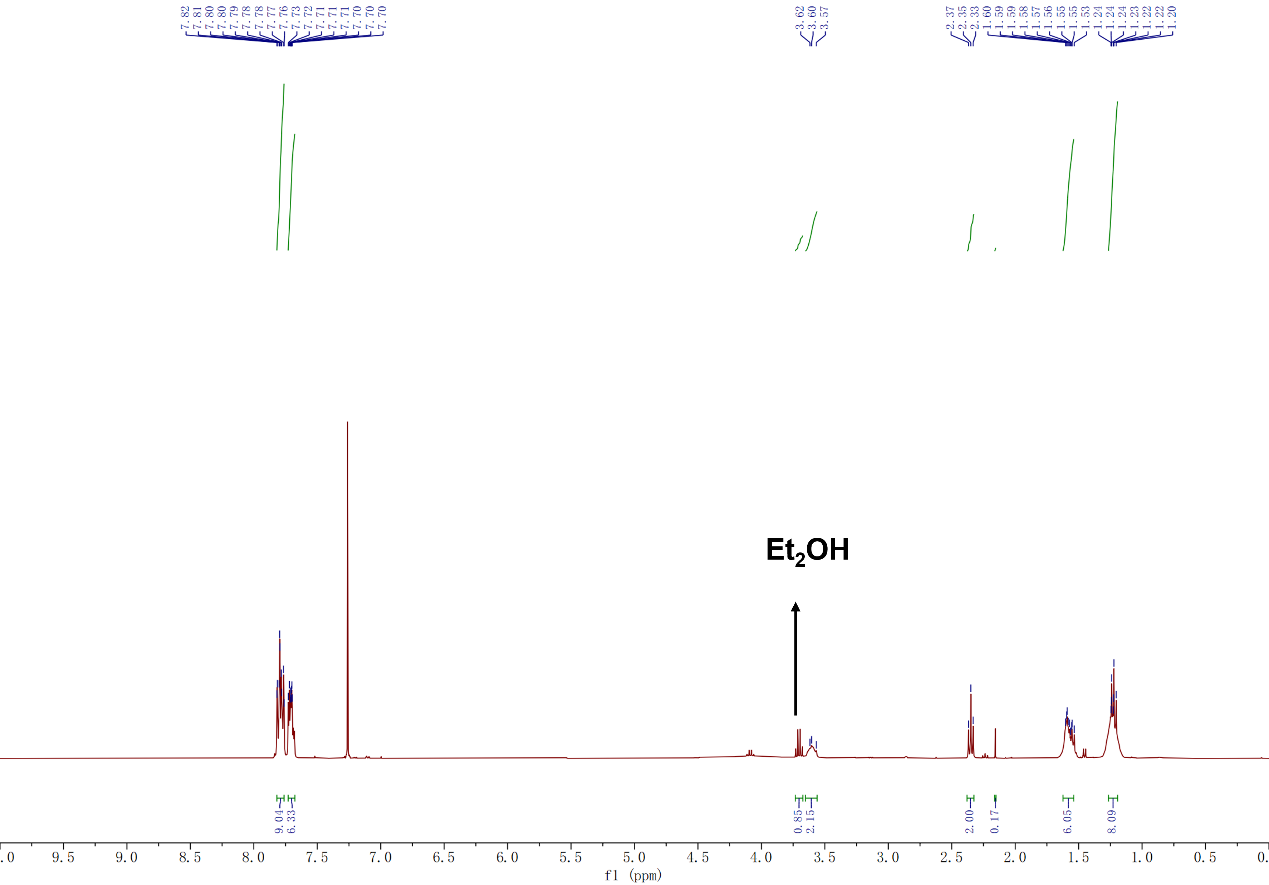


**Figure S23.** Comparison of the ^1^H NMR spectra of the modular adhesive **A3** in its initial state and after 6 recovery cycles.

**Figure S24.** Comparison for adhesion performance with previously reported adhesives on **PTFE** substrate.

| **Types** | **Material** | **substrates** | **Adhesive Strength (Adhesion strength in air at RT)** |
| --- | --- | --- | --- |
| IL-based molecular solids | P2 | Steel  PET  PTFE | 4.174 MPa  NA  0.277MPa |
| Small molecular supramolecular pseudoeutectic adhesives | 6-HTPB | Steel  PET  PTFE | 2.07 MPa  1.06 MPa  NA |
| Supramolecular Adhesives | P-DMS-Pt-H | Steel  PET  PTFE | 1.25 MPa  NA  NA |
| Low-molecular-weight supramolecular adhesive | OE | Steel  PET  PTFE | 0.55 MPa  NA  0.41 MPa |
| Aqueous co-assembly of basic amino acid monomers and polyoxometalates | His/SiW | Steel  PET  PTFE | 0.032 MPa  0.36 MPa  NA |
| Low-Molecular-Weight Supramolecular Adhesives | P1 | Steel  PET  PTFE | 4.174 MPa  NA  0.277 MPa |
| Deep Eutectic Supramolecular Polymers | BM-2 | Steel  PET  PTFE | 6.57 MPa  NA  NA |
| Low-molecular-weight supramolecular adhesives | C_12_TAB/ChCl | Steel  PET  PTFE | 172.6 ± 26.1 kPa  NA  127.4 ± 6.5 kPa |
| Supramolecular Adhesives | PEG−TA HMSG | Steel  PET  PTFE | 4.7 MPa  0.5 MPa  0.2 MPa |
| Supramolecular polymers | PC_10_-W_1_ | Steel  PET  PTFE | 4.28 MPa  NA  0.09 MPa |
| Poly(organosiloxane)-based adhesives | P(DPOP-Si) | Steel  PET  PTFE | 1.6 MPa  0.3 MPa  0.2 MPa |
| PAA-based adhesive | SMP_0.9_GNs_0.1_ | Steel  PET  PTFE | 6.02 MPa  0.35 MPa  0.29 MPa |
| PAA-based adhesive | PVA_1_/PAA_1.5_450k_ | Steel  PET  PTFE | 5.7 MPa  NA  NA |
| IL-based supramolecular aggregates | P1-BF4 | Steel  PET  PTFE | **3.95 MPa**  **0.58 MPa**  **0.51 MPa** |
| PSA-PUA adhesive | PSA-PUA20 | Steel  PET  PTFE | **134.34 kPa**  **235.96 kPa**  **137.70 kPa** |
| Polymer adhesive | ANA-5 | Steel  PET  PTFE | **NA**  **1.05 MPa**  **NA** |
| Polymer adhesive | CTP-2 | Steel  PET  PTFE | **3.5 MPa**  **0.75 MPa**  **0.25 MPa** |
| Polymer adhesive | PAPA_0.8_-PAA_1.2_ | Steel  PET  PTFE | **NA**  **123 KPa**  **87 KPa** |
| Polymer adhesive (chemical adhesion) | SiPUA | Steel  PET  PTFE | **NA**  **7.73 MPa**  **NA** |
| Polymer adhesive | ANA-5 | Steel  PET  PTFE | **NA**  **2.8 MPa**  **NA** |
| Polymer adhesive (chemical adhesion) | B11 | Steel  PET  PTFE | **NA**  **11.05 MPa**  **NA** |
| Our Work | A3 | Steel  PET  PTFE | **6.95 MPa**  **4.60 MPa**  **0.49 MPa** |

**Table S3.** Comparison for adhesion performance with previously reported adhesives.

**Figure S25.** Molecular structures of hydrogen bond acceptors (HBAs).

**Figure S26.** Adhesion strength of eutectogels prepared with different HBAs (measured immediately after bonding).


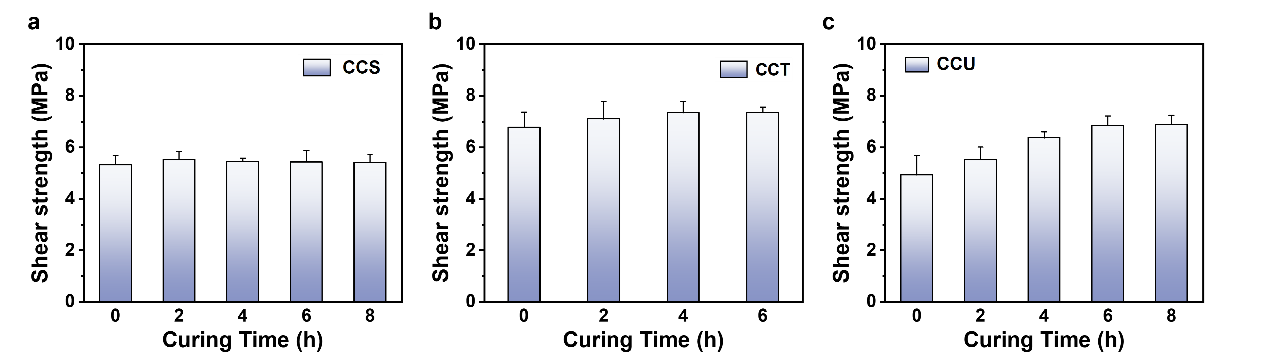


**Figure S27.** Time-dependent adhesion strengths of CCS (a), CCT (b) and CCU (c) adhesive on Steel. (RH = 20%, 25 °C).

**Figure S28.** Lap-shear strength of CCT adhesives based on the different weight ratios after curing for 6 hours (**A3**: ChCl: Thiourea).

**Figure S29.** Component necessity verification (measured immediately after bonding).

**Figure S30.** FT-IR spectra of **A3**, ChCl, Thiourea and CCT adhesive.


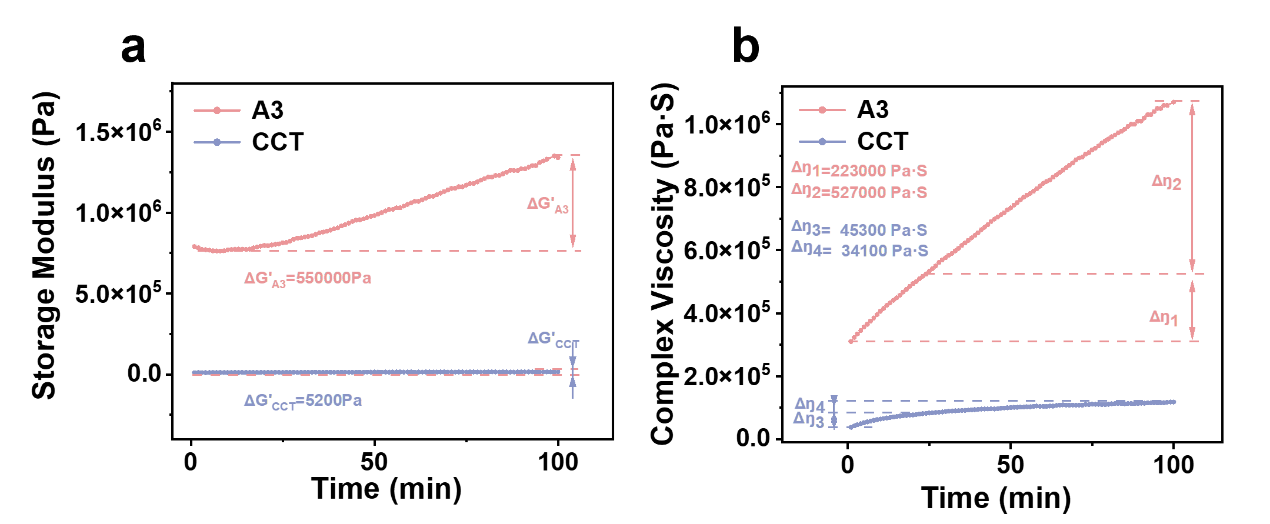


**Figure S31** Time-dependent rheological behavior of CCT and **A3**: (a) Storage modulus (G′) during curing; (b) Time-resolved viscosity profiles.

**Figure S32.** DSC curve measured by CCT adhesive which is completely cured.

| Reference | Adhesion on PET (MPa) | Reversible Cycles | Curing Time | Substrate universality  (hydrophilic substrates; hydrophobic substrates) | Solvent Resistance |
| --- | --- | --- | --- | --- | --- |
| Angew. Chem. Int. Ed. **2024**, *63*, e202403220. | NA | 10 | 48 h | 3.42-7.6 MPa;  <= 0.82 MPa | NA |
| Adv. Funct. Mater. **2025**, 2502134. | 0.58 | 10 (Underwater) | 12 h | 2.58-8.37 MPa;  <=0.58 MPa | Water |
| Sci. Adv. **2022**, 8, eadd8527. | 0.3 | 100 | Pressing and drying | 1.4-3.5 MPa;  <=0.3 MPa | pH values of 1 to 14 and different saltsolutions |
| Green Chem. **2023**, 25, 6845–6852. | 1.06 | 3 | N/A | 2.1-2.77 MPa;  =1.06 MPa | Dioxane, ethyl acetate, petroleum ether, toluene |
| **This work (A3)** | **4.6** | **8** | **24 h**  **Instantaneous adhesion with DES** | **3-6.95 MPa;**  **<=4.6 MPa** | **Toluene, ethyl acetate, ether, petroleum ether, silicone oil** |

**Table S4.** Comparison of PET adhesion strength, reusability, curing time, substrate versatility, and solvent resistance among representative small-molecule adhesives.

**Supplementary References**

[1] E. Torres, G. A. DiLabio, *J. Phys. Chem. Lett.* **2012**, *3*, 1738–1744.

[2] A. R. Lewis, K. P. Reber, *Tetrahedron Letters* **2016**, *57*, 1083–1086.

[3] A. K. Migglautsch, M. Willim, B. Schweda, A. Glieder, R. Breinbauer, M. Winkler, *Tetrahedron* **2018**, *74*, 6199–6204.

[4] S. Thurnhofer, W. Vetter, *Tetrahedron* **2007**, *63*, 1140–1145.
